# Xolography for Rapid Volumetric Production of Objects from the Nanoscopic to Macroscopic Length Scales

**DOI:** 10.1002/adma.202503245

**Published:** 2025-06-29

**Authors:** Xichuan Li, Yuan Xiu, Kenny Lee, Jin Zhang, Nathaniel Corrigan, Cyrille Boyer

**Affiliations:** ^1^ Cluster for Advanced Macromolecular Design School of Chemical Engineering University of New South Wales Sydney NSW 2052 Australia; ^2^ School of Mechanical and Manufacturing Engineering University of New South Wales Sydney NSW 2052 Australia; ^3^ Australian Centre for Nanomedicine University of New South Wales Sydney NSW 2052 Australia

**Keywords:** auxiliary‐free fabrication, high resolution, nanostructure, polymerization‐induced microphase separation, self‐assembly, volumetric 3D printing, xolography

## Abstract

Light‐mediated 3D printing has revolutionized additive manufacturing, progressing from pointwise stereolithography, to layer‐by‐layer digital light processing, and most recently to volumetric 3D printing. Xolography, a novel light‐sheet‐based volumetric 3D printing approach, offers high‐speed and high‐precision fabrication of complex geometries unattainable with traditional methods. However, achieving nanoscale control (<100 nm) within these 3D printing systems remains unexplored. This work leverages polymerization‐induced microphase separation (PIMS) within the xolography process to prepare network polymer materials with simultaneous control over feature sizes at the nano‐, micro‐, and macro‐scale. By controlling the chain length and mass fraction of macromolecular chain transfer agents used in the PIMS process, precise manipulation of nanodomain size within 3D printed materials is demonstrated, while optimization of the other resin components enables the fabrication of rigid materials with feature sizes of 80 µm. Critically, the rapid one‐step fabrication of complex and multi‐component structures such as a functional waterwheel with interlocking parts, at high volume‐building rates is showcased. This combined approach expands the design space for functional nanomaterials, opening new avenues for applications in diverse fields such as polymer electrolyte membranes, biomedical delivery systems, and semi‐permeable microcapsules.

## Graphical abstract

This article explores the development of materials with multi‐scale control at the nanoscale (<100 nm), micrometer‐scale (80 µm), and macro‐scale (multi‐component geometries). This is achieved by applying polymerization‐induced microphase separation (PIMS) process via volumetric 3D printing (Xolography), expanding the design space for functional nanomaterials in diverse applications.

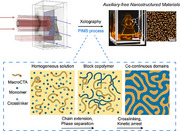

## 1 Introduction

Light‐based 3D printing has garnered significant attention for its ability to fabricate intricate geometries with high precision and spatial control using eco‐friendly light sources. This technology has enabled the development of functional materials with broad applications, including sensing,^[^ ^1^ ^]^ actuation,^[^ ^2^ ^]^ biomaterials,^[^ ^3^ ^]^ and beyond. Among various light‐based techniques, stereolithography and digital light processing,^[^ ^4^ ^]^ are particularly well‐established and widely adopted methods for fabricating customized objects. Stereolithography excels at achieving high resolution and intricate details through precise laser tracing, but its point‐by‐point curing process limits the build speed. Conversely, digital light processing employs projected light patterns to cure entire layers simultaneously, offering faster build speeds. However, its layer‐by‐layer approach can produce visible “stair‐stepping” artifacts on curved or angled surfaces,^[^ ^5^ ^]^ particularly if layer adhesion and resin recoating are not optimized. Continuous liquid interface production aims to address some of these challenges by enabling continuous fabrication without resin recoating, but its reliance on a build platform restricts geometric freedom and material viscosity.^[^ ^6^ ^]^ Additionally, these techniques usually require the use of auxiliary supports for overhanging features, which results in another downstream processing step to remove the supports.^[^ ^5^, ^7^ ^]^

In this context, emergent volumetric 3D printing technologies offer a transformative alternative by enabling non‐contact, support‐free fabrication within the entire resin volume.^[^ ^8^ ^]^ This eliminates the need for support structures and detachment processes, which can damage delicate objects. Furthermore, volumetric printing opens a new avenue for fabricating complex, multicomponent objects, thereby broadening the possibilities of additive manufacturing. Among volumetric techniques, two‐photon polymerization,^[^ ^9^ ^]^ computed axial lithography,^[^ ^10^ ^]^ and light‐sheet‐based 3D printing,^[^ ^11^ ^]^ including xolography,^[^ ^12^ ^]^ have emerged as leading approaches. Of these, two‐photon polymerization offers unmatched resolution, achieving features as small as 65 nm by exploiting the nonlinear nature of two‐photon absorption to overcome the diffraction limit.^[^ ^13^ ^]^ Nevertheless, this high precision comes at the cost of speed, with fabrication rates typically under 20 mm^3^ h^−1^.^[^ ^14^ ^]^ In contrast, computed axial lithography enables ultrafast solidification by curing an entire volume of liquid resin in a single step, fabricating centimeter‐scale objects within 30 to 120 s.^[^ ^10^ ^]^ Computed axial lithography achieves this by superimposing 2D images projected from multiple angles, solidifying the resin within the high‐dose 3D profile while leaving other low‐dose regions unsolidified below the gelation threshold. Despite its speed, this technique typically suffers from limited resolution due to light scattering as it passes through the solidifying resin. The need to incorporate inhibitors to prevent undesired polymerization further adds complexity. Even with optimization, computer axial lithography achieves a resolution of ≈80 µm for positive features and 500 µm negative ones.^[^ ^15^ ^]^ While recent advancements have reduced minimum feature sizes to 50 µm, this improvement often comes at the expense of a small overall printable volume.^[^ ^16^ ^]^

**Figure 1:**
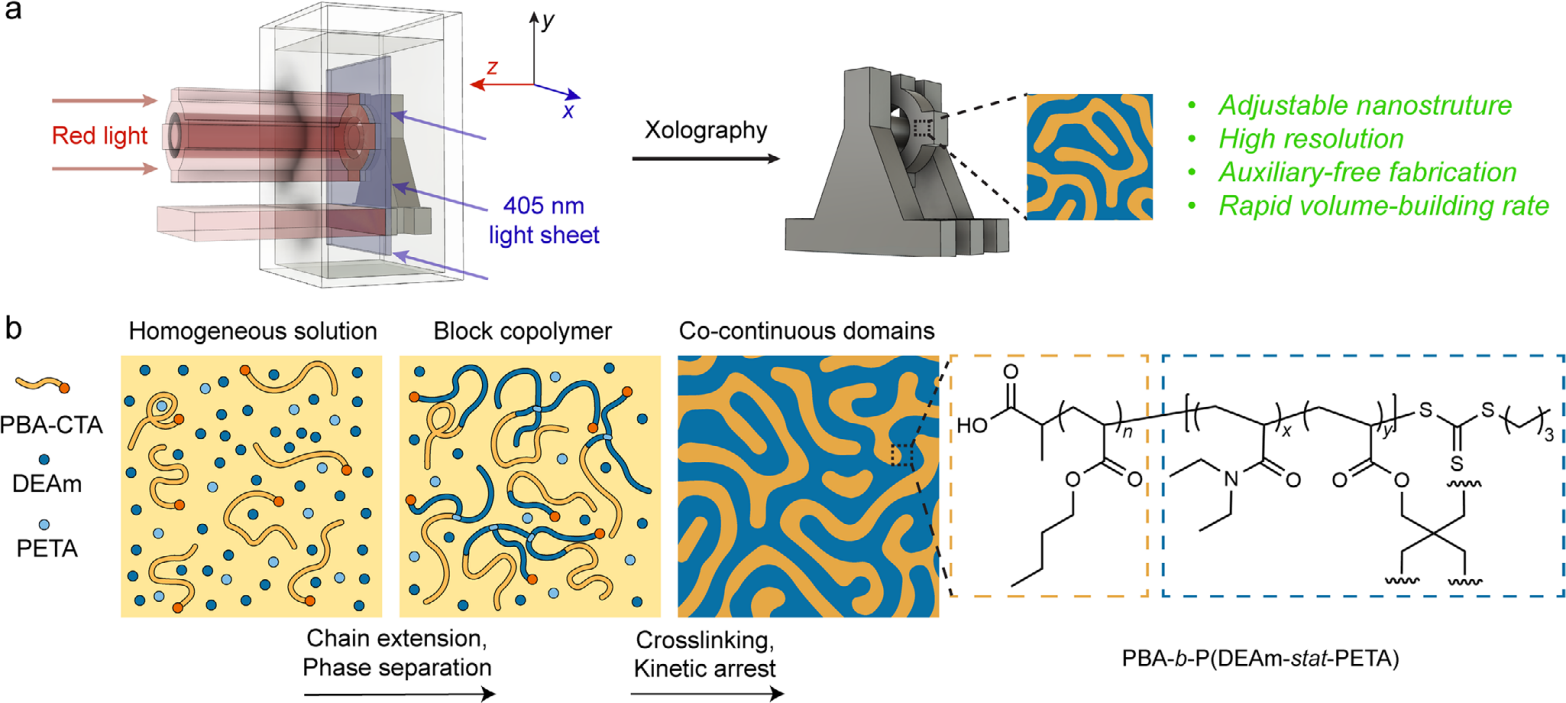
Protocols to fabricate nanostructured polymer materials via xolography. a) Volumetric production of materials exhibiting a variety of advantages in this work via polymerization‐induced microphase separation. b) Schematic illustration of polymerization‐induced microphase separation process: a macroCTA (PBA‐CTA) is chain extended with *N,N*‐diethylacrylamide (DEAm) and pentaerythritol tetraacrylate (PETA) to form PBA‐*b*‐P(DEAm‐*stat*‐PETA) network with nanodomains due to the thermodynamic incompatibility between PBA‐CTA and P(DEAm‐*stat*‐PETA) blocks.

Xolography has recently emerged as a promising alternative volumetric 3D printing technique, offering superior resolution compared to computed axial lithography, while achieving high volume‐building rates of up to 198 000 mm^3^ h^−1^, ≈10^4^ times faster than two‐photo polymerization.^[^ ^12^ ^]^ Xolography employs a dual‐color printing technique, where two different light sources radiate the resin simultaneously (**Figure** **1**a). Polymerization occurs only at the intersection of both wavelengths of light, whereas areas exposed to a single wavelength of light remain unsolidified. The resolution in these processes has been demonstrated to be as low as 20 µm for positive features and 100 µm for negative ones.^[^ ^17^ ^]^ However, the adoption of xolography remains limited by the narrow range of compatible resins,^[^ ^12^, ^17^, ^18^ ^]^ as resins must exhibit high viscosity, rapid reactivity, and high transparency to meet the stringent demands of the two‐step photochemical activation (vide infra). Despite these challenges, xolography has demonstrated excellent control over micrometer‐ and centimeter‐scale structures, however, achieving nanoscale structures via xolography (<100 nm) has never been reported.

The current work extends xolography to the nanoscale by developing, applying, and optimizing resins designed for polymerization‐induced microphase separation (PIMS).^[^ ^19^ ^]^ PIMS, unlike its macroscopic counterpart polymerization‐induced phase separation (PIPS),^[^ ^20^ ^]^ precisely controls nanostructure formation by employing macromolecular chain transfer agents (macroCTAs) prepared via a living polymerization technique, such as reversible addition‐fragmentation chain transfer (RAFT) polymerization. During the PIMS process, these macroCTAs are chain‐extended with specific monomers and crosslinkers (Figure 1b), which triggers thermodynamically driven microphase separation. This process yields globally disordered but locally ordered nanostructured materials with domain sizes below 100 nm. Importantly, these small domain sizes ensure the high optical transparency required for visible light‐mediated xolography. Furthermore, the size and morphology of the nanodomains can be readily tuned by adjusting the molecular weight, mass fraction and architecture of macroCTAs.^[^ ^21^ ^]^ As a result, the PIMS process has been applied to develop nanoporous materials,^[^ ^22^ ^]^ polymer electrolytes,^[^ ^23^ ^]^ and heterogeneous catalysts.^[^ ^24^ ^]^ In this work, we formulated a suite of PIMS resins optimized for xolography using poly(*n*‐butyl acrylate) as macroCTA (PBA‐CTA), *N*,*N*‐diethyl acrylamide (DEAm) as monomer and pentaerythritol tetraacrylate (PETA) as crosslinker. Characterization via small‐angle X‐ray scattering (SAXS) and atomic force microscopy (AFM) revealed tunable domain spacing (18–66 nm) and a range of morphologies, including globular, elongated, and bicontinuous phases. Furthermore, we successfully fabricated auxiliary‐free multicomponent objects in a single pass using viscous PIMS precursors. These advancements enable the creation of complex geometries inaccessible to conventional 3D printing techniques, coupled with rapid fabrication speeds (up to 21 360 mm^3^ h^−1^). In comparison, the maximum print rate used in this work is 5.93 mm^3^ s^−1^, and the minimum voxel size is 80 µm, indicating the total peak printing rate is 1.16 × 10^4^ voxels/s for this light‐sheet 3D printing technique.^[^ ^9b^ ^]^ In general, high‐resolution feature sizes and high fabrication rates for diverse functional materials are required yet deemed as common challenges in additive manufacturing.^[^ ^25^ ^]^ Apart from resolving these issues, this approach also simplifies post‐treatment processes and removes potential damage to delicate samples during detachment. The PIMS‐based xolography method presented in this study holds significant promise for a range of applications, including catalysis,^[^ ^24^ ^]^ biomedical delivery,^[^ ^26^ ^]^ and energy storage.^[^ ^27^ ^]^

## 2 Results and Discussion

### 2.1 Key Requirements for the Fabrication of PIMS Materials via Xolography

**Figure 2:**
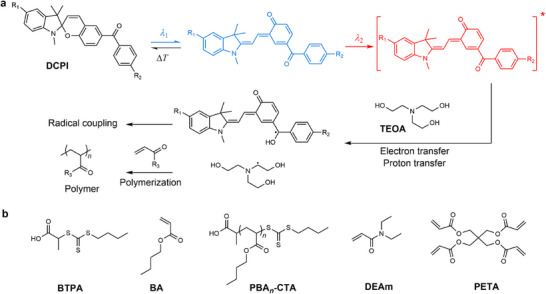
Proposed mechanism of photoinitiation in xolography and all compounds used in this work. a) Type II photoinitiation of the dual‐color photoinitiator (DCPI) and the co‐initiator (TEOA), which goes through electron and proton transfer processes to form free radicals for polymerization. b) Chemical structures of resin constituents.

Xolography utilizes a dual‐color photoinitiator (DCPI) and an orthogonal light projection system. This system combines a polarized laser‐based violet light sheet (405 nm) with a visible light source (referred to as red light) from a digital light projector, emitting across a broad spectrum from 540 to 720 nm (Figure S1, Supporting Information). For 405 nm light sheet, the optical information was characterized by a camera sensor as shown in Figure S2 (Supporting Information). Figure S2a (Supporting Information) shows the partial image of 405 nm light sheet in one arm at the waist position, through which the light intensity distribution was measured as shown in Figure S2b (Supporting Information). In addition, the light sheet thickness varies alongside the optical pathway indicated as the beam waist (≈50 µm, Figure S2c, Supporting Information). The beam waist tends to widen toward the edges of the print vat from the center. This is due to the transformation of a Gaussian beam from a 405‐nm diode laser into a diverging laser line, which is subsequently collimated and focused into the center of the print volume.^[^ ^12^ ^]^ Both the DCPI and the orthogonal light projection system are crucial in triggering photopolymerization and controlling the geometries of target 3D‐printed objects. DCPI, consisting of a spiropyran photoswitch with a covalently connected but masked benzophenone moiety, is used to initiate polymerization (**Figure** **2**a). Specifically, violet light facilitates the unmasking of the benzophenone group by ring opening of the spiropyran to form a metastable merocyanine; this merocyanine can spontaneously revert to the stable spiropyran form by thermal relaxation. Alternatively, if the metastable merocyanine is exposed to red light, the unmasked benzophenone moiety of DCPI, in combination with a co‐initiator (tertiary amine), can generate free radicals to initiate polymerization. Based on this mechanism, the geometries of 3D printed objects are precisely controlled by the dual‐wavelength orthogonal‐projection system (Figure 1a). During xolography, a thin layer of DCPI molecules within the violet light sheet is activated to form metastable merocyanine, which leads to the formation of carbon‐centered radicals under red light irradiation from a perpendicular projector focusing on the model's cross‐sectional images (Figure 2a). In principle, only the resin at the intersection of the two lights is solidified,^[^ ^12^ ^]^ with the target object fabricated through the continuous, synchronized movement of the violet light sheet and the dynamic variation of cross‐sectional images projected via the red‐light source. This innovative dual‐color approach enables the rapid, auxiliary‐free fabrication of objects with high volumetric throughput.

Based on the distinct operating system of xolography, several key factors must be considered to formulate a resin system that can fabricate objects with high qualities and at fast speeds. First, the resin mixture must exhibit complete miscibility and excellent optical transparency to ensure uniform violet light irradiation along the optical light‐sheet path within the resin, leading to isotropic 3D‐printed objects. Specifically, poor miscibility leads to cloudiness, scattering the incident light and creating gradients in the intensity of the 405 nm light sheet along the *x*‐direction. These intensity variations result in non‐uniform polymerization rates during xolography. Similarly, light‐absorbing compounds in the resin can also cause significant light attenuation along the optical path, further hindering uniform polymerization. Second, to mitigate the sedimentation of printed parts due to gravity, the use of a highly reactive photoresist combined with a highly viscous resin is essential. This combination facilitates rapid solidification of the liquid resin, significantly reducing distortion caused by sedimentation during the 3D printing process, which typically completes within a few minutes for xolography. In this case, an efficient initiation system is essential to achieve high conversion of double bonds, ultimately imparting the printed object with sufficient rigidity for facile post‐treatment.

Beyond the general parameters for xolography, achieving nanostructured materials via PIMS introduces further constraints on the resin formulations. Typical PIMS systems employ a macroCTA that is chain extended with a monomer and a crosslinker, forming macroCTA‐*b*‐P(monomer‐*stat*‐crosslinker) diblock copolymers (Figure 1b). Microphase separation occurs during polymerization and is driven by the thermodynamic incompatibility between these two blocks, in turn directing the self‐assembly of the polymers. Ultimately, the nanostructured materials are kinetically trapped as the crosslinker forms a network copolymer. Therefore, the incompatibility between the two blocks, quantified by segregation strength *χN*, is a critical parameter for achieving nanostructured materials via PIMS‐based xolography.

### 2.2 Optimization of Resin Formulations

Based on the aforementioned requirements, a series of resins were formulated to assess their suitability for application to xolography. Poly(*n*‐butyl acrylate) capped with a RAFT‐agent (PBA*_n_*‐CTA) was selected as macroCTA block due to its low glass transition temperature (*T*_g_)and hydrophobicity, which promotes the formation of soft nano‐domains.^[^ ^28^ ^]^ Specifically, PBA*_n_*‐CTA was synthesized via RAFT polymerization, using *n*‐butyl acrylate (BA) as monomer, 2‐(*n*‐butylthiocarbonothioylthio) propanoic acid (BTPA) as RAFT agent, and 2,2’‐azobis(2‐methylpropionitrile) (AIBN) as thermal initiator with the reaction conducted at 60 °C (Figure S3, Supporting Information). A series of PBA*_n_*‐CTAs with varying degrees of polymerization (*X*_n_) were synthesized and characterized. ^1^H NMR spectroscopy (Figures S4 and S5, Supporting Information) was used to determine their *X*_n_, while gel permeation chromatography (GPC, Figure S6, Supporting Information) was employed to obtain molecular weight distributions and dispersity (*Ð*, Table S1, Supporting Information). The *X*_n_ of the PBA*_n_*‐CTAs used in this work were 96, 182, 332, and 500. To induce phase separation, *N,N*‐diethylacrylamide (DEAm) and pentaerythritol tetraacrylate (PETA) were selected as monomer and crosslinker, respectively. This combination effectively solubilized the PBA*_n_*‐CTAs, providing visibly transparent solutions that also exhibited rapid polymerization rates and the potential of microphase separation when using PBA*_n_*‐CTAs of sufficient chain length. For PBA‐*b*‐P(DEAm‐*stat*‐PETA), the calculated *χ* is 0.1825 (Equation S9, Supporting Information), indicating that a total degree of polymerization (*N*) exceeding 55 is necessary to achieve adequate microphase separation, based on the requirement that *χN* > 10.^[^ ^29^ ^]^

For the photoinitiation system (DCPI and co‐initiator), different amine‐based co‐initiators were investigated to ensure rapid and sufficient solidification during volumetric 3D printing. While tributylamine and *N*‐methyldiethanolamine provided homogeneous resins and enabled the generation of solid materials within relatively short timeframes, triethanolamine exhibited the fastest gelation. This was attributed to the higher initiation efficiency of hydroxyalkyl tertiary amines in type II photoinitiation compared to their alkyl tertiary amine counterparts (Table S2, Supporting Information).^[^ ^30^ ^]^ Consequently, triethanolamine was selected as co‐initiator for subsequent experiments.

After confirming the resin constituents, we investigated the effects of varying their relative concentrations. The loading of PBA*_n_*‐CTA was adjusted from 10 to 40 wt%, allowing for nanostructure control by tuning the volume fraction of each block. To evaluate the chain extension efficiency with different photoinitiation systems, PBA*_n_*‐CTA was chain‐extended with DEAm in the absence of crosslinker, using various mass concentrations of TEOA and DCPI (Table S3, Supporting Information). As shown in Figure S7 (Supporting Information), increasing the concentrations of TEOA and DCPI improved chain extension efficiency. However, higher DCPI concentrations caused significant light‐intensity gradients within the print resin. This phenomenon was demonstrated through theoretical curves (Figure S10 and Table S4, Supporting Information) that compared the light intensity across the resin vat (a cuvette) at different PBA*_n_*‐CTA and DCPI concentrations. Since both DCPI and PBA‐CTA absorb at 405 nm (Figures S8 and S9, Supporting Information), high loadings of low *X*_n_ PBA*_n_*‐CTA or DCPI significantly reduced the 405 nm light intensity at the center of the cuvette. Balancing the need for efficient chain extension of the macroCTA with the desire to minimize light absorption effects, mass fractions of 5 wt% TEOA and 0.05 wt% DCPI were selected for subsequent 3D printing. Higher TEOA concentrations (>5 wt%) were also avoided to prevent excessive plasticization of the 3D‐printed materials. The light absorbance of 0.05 wt% DCPI (≈0.16, Figure S8, Supporting Information) used in this work is within the linear absorbance range (Figure S9a, Supporting Information).

Achieving a balance between DEAm and PETA concentrations is also crucial for allowing sufficient time for microphase separation before kinetic arrest by crosslinking and providing adequate crosslinking points for rapid solidification. To determine an optimal ratio, two DEAm:PETA mass ratios (1.6:1 and 3.3:1) were tested to fabricate objects via xolography for resolution tests, using 30 wt% PBA_332_, 5 wt% TEOA, and 0.05 wt% DCPI. As shown in Figure S11 (Supporting Information), both formulations successfully printed fine details, with the positive features being slightly clearer for the 3.3:1 system (60 µm), while both systems showed fine negative features (80 µm). Based on these results, and considering the high print resolution achieved with these unoptimized systems, an intermediate DEAm:PETA mass ratio of 2:1 was selected for the rest of this work. With the DEAm:PETA mass ratio and the concentrations of DCPI and TEOA fixed, the remaining variable parameters for the resin formulations are the PBA*_n_*‐CTA *X*_n_ and the PBA*_n_*‐CTA loading. Thus, the resins are named using the convention MXXX‐YY, where XXX represents the PBA*_n_*‐CTA *X*_n_ and YY represents the PBA*_n_*‐CTA loading in wt% (Table S5, Supporting Information). For example, the resin M500‐30 contains 30 wt% of the PBA_500_‐CTA, 65 wt% of 2:1 w/w DEAm:PETA, 5 wt% of TEOA and 0.05 wt% DCPI (the latter being considered negligible in the total mass calculation).

### 2.3 Optimization of Printing Conditions

Having established the resin components and their concentration ranges, a matrix of resins was prepared and 3D printed at varying xolography print speeds and 405 nm laser light‐sheet intensities. This optimization aims to assess the impact of the xolography printing conditions on both the ability to print materials and their resulting feature resolution. As shown in Equation S13 (Supporting Information), the energy dose is proportional to the ratio of laser light‐sheet intensity to printing speed. This energy dose directly influences the curing efficiency of the liquid resins. Excessive energy dose can lead to over‐curing due to unwanted single‐wavelength (405 nm) activation of DCPI and subsequent radical generation,^[^ ^12^ ^]^ thereby reducing the geometric precision of the digital light projector. Conversely, insufficient energy doses lead to only partially cured materials that lack mechanical integrity. Only within an appropriate energy dose range can the object be precisely printed with well‐defined features.

**Figure 3:**
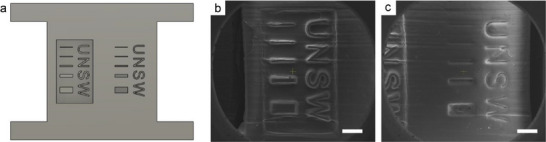
SEM micrographs of the object printed from the M332‐40 resin. a) The model was designed for a resolution test, with the left area indicating positive (protruding) features and the right one denoting negative (sunken) features. The lateral widths of lines from top to bottom were designed to be 20, 40, 80, 160, and 320 µm, respectively. The printed object showcases b) positive and c) negative features. The scale bars are 500 µm.

The printing process was optimized by starting at the highest laser light‐sheet intensity (12.8 mW mm^−2^) and a high printing speed (0.8 mm min^−1^), then systematically decreasing both parameters until the optimal resolution of the printed material was achieved. For this analysis, a specially designed test piece that featured elevated and sunken lettering of various sizes, along with calibration lines with target widths of 20, 40, 80, 160, and 320 µm, was employed (**Figure** **3**a). Resolution was first determined by optical microscopy and further analyzed via scanning electron microscopy (SEM). Printing speed was maintained as high as possible to maximize build rates. The optimization process is exemplified using M500‐30 formulation (Figure S12, Supporting Information), which shows optical micrographs of positive features, printed at various light intensities but at a constant speed of 0.8 mm min^−1^. At the highest light intensity (9.6 mW mm^−2^), fine details of the printed sample appeared blurred, with significantly thinner outlines than in the digital model due to overcuring (Figure S12c, Supporting Information). Improved fidelity of positive features was observed upon decreasing light intensity to 4.8 mW mm^−2^ and further to 2.4 mW mm^−2^ (Figure S12a,b, Supporting Information). Subsequent SEM analysis confirmed the resolution, revealing well‐defined features in the materials printed at 2.4 mW mm^−2^ and 0.8 mm min^−1^ (Figure S13, Supporting Information). A similar optimization procedure was performed for the other resins, systematically adjusting either the speed or the intensity to identify the optimal print conditions, as determined by optical microscopy.

Following the optimization, all samples were examined by SEM to assess feature resolution under their respective curing conditions (Figures S14 and S15, Supporting Information). Quantitative resolution assessment was conducted using a column of calibrated lines with widths of 20, 40, 80, 160, and 320 µm to check the highest resolution of objects fabricated with PIMS resins. For all systems, well‐defined details for the 20 and 40 µm samples were not observed under the current conditions, which provides the upper limit of feature fidelity using the resins developed in this work. Due to this limitation, samples were classified as having good print resolution if, based on SEM imaging, the line widths are within ± 10% of their target values for the 80,160 and 320 µm lines (Tables S6 and S7, Supporting Information). One of the high‐resolution objects is exemplified in Figure 3, printed from M332‐40. The positive features were clearly defined (Figure 3b), while the negative characters, though less conspicuous, still exhibited good resolution at the 80 µm calibration line and above (Figure 3c).

Figures S14 and S15 (Supporting Information) show SEM images focused on the positive and negative UNSW features for all optimized conditions. Notably, some low‐resolution samples were produced from resins with low macroCTA mass loading or short‐chain‐length PBA*_n_*‐CTA, primarily due to their lower viscosities, as higher viscosity mitigates sedimentation of solidified parts during xolography. More specifically, the difference in density between the polymerized material and the starting resin can lead to object sedimentation and a subsequent loss of resolution.^[^ ^17a^ ^]^ Employing high‐viscosity resins and rapid printing speeds can minimize this undesirable effect. For our systems, viscosity measurements (Table S8, Supporting Information) indicate that well‐defined geometries were achieved for resins with viscosities exceeding 73 mPa s.

**Table** **1** reveals a clear trend: the optimal energy dose for xolography decreases as the PBA*_n_*‐CTA chain length (*X*_n_) increases at constant loading. This is attributed to the lower concentration of BTPA end‐groups at higher *X*_n_, reducing light attenuation along the 405 nm optical pathway (Table S9, Supporting Information). In addition, resin viscosity increases significantly at higher PBA*_n_*‐CTA *X*_n_, restricting the mobility of propagating chains and thus leading to reduced termination (Table S8, Supporting Information).^[^ ^31^ ^]^ These combined factors contribute to the lower 405 nm energy dose required for resins with higher *X*_n_. A lower light dose suppresses unwanted single‐wavelength (405 nm) initiation, enhancing cross‐sectional shape control by the red light from the orthogonal digital light projector. Notably, M96‐40, despite exhibiting the largest light deviation between the side and middle (30.4%), still produced high‐resolution prints, indicating that xolography can tolerate moderate light attenuation (Table S4, Supporting Information). Beyond the factors discussed above, another critical parameter influencing the xolography process is the half‐life of DCPI (Figure S16, Supporting Information), which governs the thermal relaxation of metastable merocyanine back to its dormant spiropyran state. Extended DCPI half‐lives result in a considerable number of merocyanine molecules with active benzophenone moieties that are outside of the laser light sheet, causing unwanted radical generation and loss of shape control during the short print period. All resin formulations used in this work showed DCPI half‐lives ranging from 4.0 to 5.8 s, providing both efficient activation and suitable thermal relaxation of metastable DCPI back to the dormant state (Table S8, Supporting Information).

**Table 1:** Optimal printing conditions for resins tested in xolography.

Resin^a)^	Printing conditions			
	Speed [mm min^−1^]	Intensity [mW mm^−2^]	Energy dose^b)^ [mJ mm^−3^]	Resolution^c)^
M96‐10	0.6	12.8	6.4	Poor
M182‐10	0.8	12.8	4.8	Poor
M332‐10	0.8	6.4	2.4	Poor
M500‐10	0.8	6.4	2.4	Poor
M96‐20	0.6	12.8	6.4	Poor
M182‐20	0.8	4.8	1.8	Poor
M332‐20	0.8	3.2	1.2	Good
M500‐20	0.8	2.4	0.9	Good
M96‐30	0.6	12.8	6.4	Poor
M182‐30	0.8	3.2	1.2	Good
M332‐30	0.8	3.2	1.2	Good
M500‐30	0.8	2.4	0.9	Good
M96‐40	0.8	12.8	4.8	Good
M182‐40	0.8	3.2	1.2	Good
M332‐40	0.8	3.2	1.2	Good
M500‐40	0.8	2.4	0.9	Good

^a)^ The naming convention is as follows: MXXX‐YY, where XXX indicates the degree of polymerization of the PBA*_n_*‐CTA and YY indicates the loading (wt%) of PBA*_n_*‐CTA in the resin;

^b)^ Energy dose is calculated based on Equation S13 (Supporting Information);

^c)^ Resolution is determined by positive features as visualized in SEM micrographs: (Table S6, Supporting Information).

### 2.4 Investigation of Nanoscale Characteristics

After optimizing the 16 resin formulations and their respective printing conditions, the nanostructure of these 3D printed materials was investigated. For this purpose, rectangular prisms were prepared and analyzed by small‐angle X‐ray scattering (SAXS, 6 × 8 × 0.8 mm) and atomic force microscopy (AFM, 6 × 8 × 2 mm). Each material was printed using its optimized parameters (speed and intensity), followed by a 5‐min postcuring step under 405 nm irradiation. To isolate the effect of light intensity on nanostructure, three M500‐30 resin samples were printed at a constant speed (0.8 mm min^−1^), but with varying intensities (2.4, 4.8, and 9.6 mW mm^−2^), ensuring ≥90% vinyl bond conversion in all cases after postcuring (Table S8, Supporting Information). SAXS analysis of 3D printed samples revealed a consistent domain spacing (*d*_SAXS_ = 52 nm) and a locally ordered, globally disordered microphase‐separated state across all samples (Figure S17, Supporting Information). This demonstrates that, within the tested range, light intensity had a negligible impact on domain spacing, supporting the established understanding that nanostructure is primarily governed by macroCTA chain length (*X*_n_) and stoichiometry.^[^ ^21^, ^32^ ^]^

**Figure 4:**
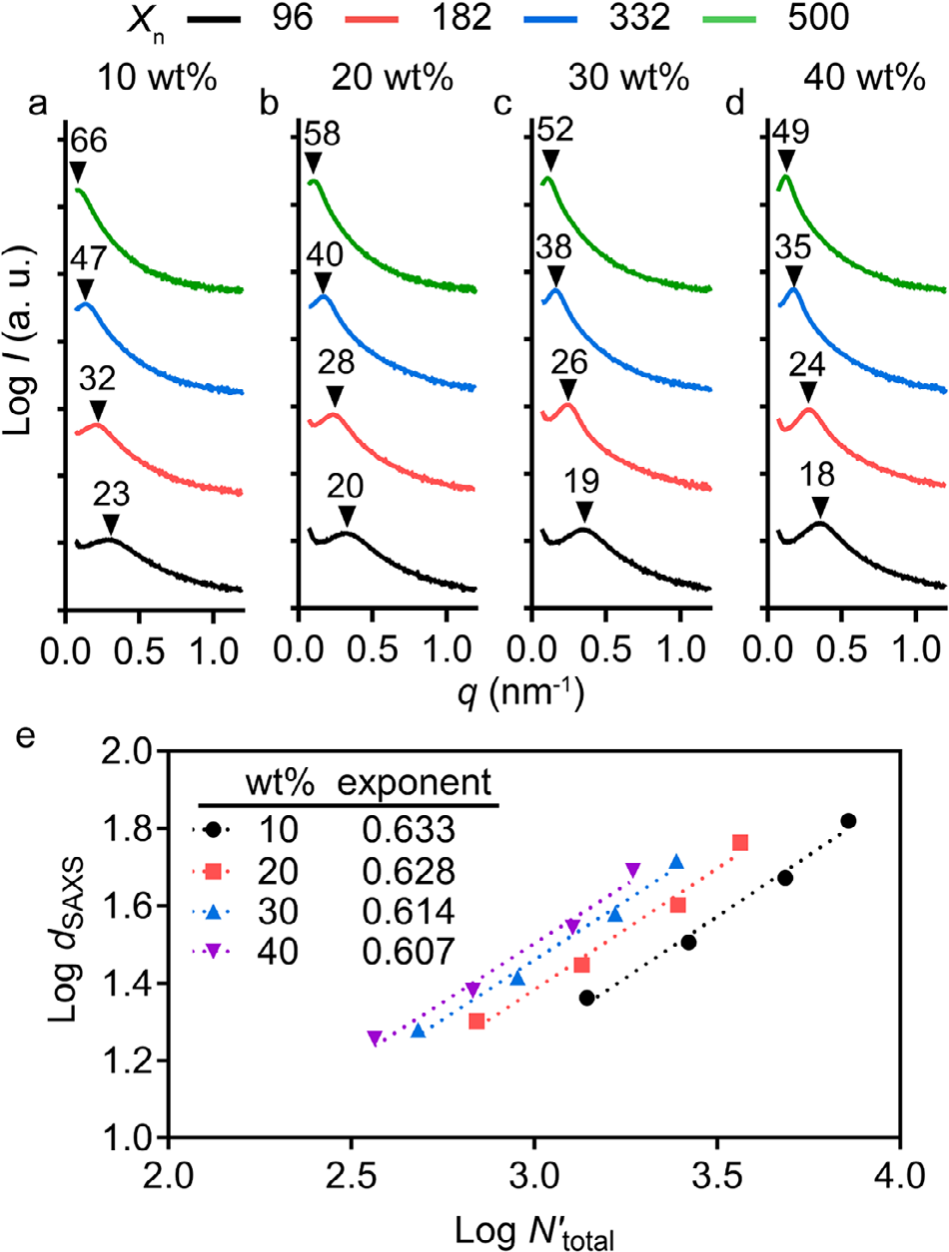
SAXS characterization of nanostructured materials prepared via xolography. Domain spacing (nm) of samples printed using a) 10 wt%, b) 20 wt%, c) 30 wt%, and d) 40 wt% PBA*_n_*‐CTA with various chain length *X*_n_, while the mass ratio of DEAm to PETA is fixed at 2:1 with 5 wt% TEOA and 0.05 wt% DCPI. Each plot is vertically shifted for clarity. e) Power law dependence of domain spacing (*d*_SAXS_) on the total volumetric degree of polymerization (*N'*_total_), where *d*_SAXS_ (nm) was obtained from SAXS results and *N'*_total_ was calculated based on a common monomer reference volume (118 Å^3^).

Subsequently, SAXS was employed to investigate the nanostructure of the PIMS materials prepared using the other resins. Each sample exhibited a single broad scattering peak, confirming a globally disordered microphase‐separated state.^[^ ^33^ ^]^ However, the peak maximum shifted to a lower *q* (larger domain spacing) as the PBA*_n_*‐CTA *X*_n_ increased from 96 to 500 (**Figure** **4**). Specifically, the domain spacing increased with higher *X*_n_, from 18–49 nm for systems with 40 wt% PBA*_n_*‐CTA and from 23 and 66 nm for those with 10 wt% PBA*_n_*‐CTA, while the systems with 20 and 30 wt% PBA*_n_*‐CTA showed intermediate domain spacing values.

The predictability of domain spacing is significant, as it directly influences material properties such as swelling and mass transport.^[^ ^34^ ^]^ Prior studies^[^ ^29^ ^]^ have shown that the domain spacing (*d*) in microphase‐separated structures follows a power law, *d* ∼ *N* ^α^, where the exponent (α) ranges from 1/2 to 2/3. At *α* = 1/2, the system is in the weak segregation limit (WSL) and polymer chains remain largely unperturbed. In contrast, α approaching 2/3 corresponds to the strong segregation limit (SSL), where chain configurations are more perturbed. As shown in Figure 4e, *d*_SAXS_ plotted against the normalized total degree of polymerization (*N'*_total_) at a fixed PBA*_n_*‐CTA loading yielded scaling exponents between 0.607 and 0.633, indicating behavior closer to SSL. The slight reduction in the exponent compared to ideal SSL behavior can be attributed to the use of PETA as a crosslinker in our resins, which restricts mobility of *net*‐P(DEAm‐*stat*‐PETA) chains during microphase separation due to kinetic arrest.

To further analyze the nanostructure of the 3D printed materials, the Teubner–Strey (T–S) model^[^ ^35^ ^]^ was employed to fit the SAXS data.^[^ ^36^ ^]^ T‐S modeling provides insights into the domain size distribution and the heterogeneity of the domain interface between PBA*_n_*‐CTA and *net*‐P(DEAm‐*stat*‐PETA) block. Three characteristic parameters were extracted via T–S model fitting: domain spacing (*d*_TS_), correlation length (*ξ*), and amphiphilicity factor (*f*_a_). *d*_TS_ represents spacing between two adjacent domains with the same composition; *ξ* describes the spatial coherence of the interfaces; *f*_a_ reflects the segregation strength at the interfaces. For *f*_a_ > 0, the corresponding material has weakly structured interfaces, while ‐1 < *f*_a_ < 0 correlates with well‐structured morphologies, and *f*_a_ = ‐1 characterizes highly ordered structures, such as those seen in perfect lamella domains.

Our modeling results revealed a strong agreement between *d*_TS_ and *d*_SAXS_ (Table S10, Supporting Information). To assess domain size dispersity,^[^ ^37^ ^]^ we analyzed the ratio of correlation length to domain spacing (*ξ/d*_TS_) across different PBA*_n_*‐CTA loadings while keeping *X*_n_ constant. Samples printed with higher PBA*_n_*‐CTA loadings exhibited increased *ξ/d*_TS_ values, indicating a narrower polydispersity. A similar trend was observed for *f*_a_, with all values below 0, confirming well‐defined morphologies and finely structured phase separation. The reduced domain size dispersity and sharper interfaces in samples with higher PBA*_n_*‐CTA loadings were further supported by the narrower SAXS peaks observed at larger PBA*_n_*‐CTA mass fractions (Figure S18, Supporting Information).

**Figure 5:**
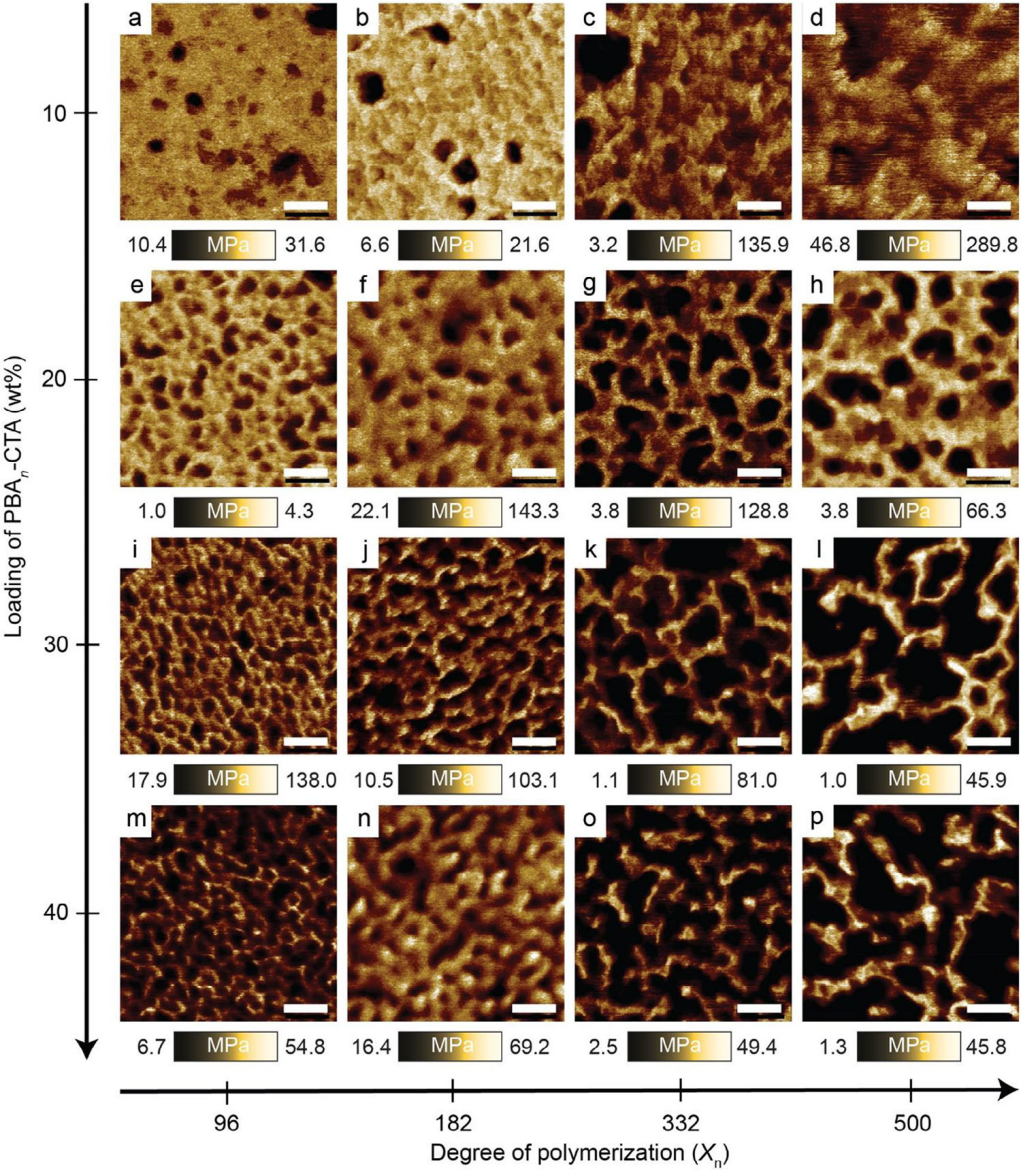
AFM characterization of nanostructured materials exhibiting a dependence of nanoscale morphologies on the chain length (*X*_n_) and loading (wt%) of PBA*_n_*‐CTA. These materials were printed using a–d) 10 wt% PBA*_n_*‐CTA with *X*_n_ = a) 96, b) 182, c) 332, d) 500; e‐h) 20 wt% PBA*_n_*‐CTA with *X*_n_ = e) 96, f) 182, g) 332, h) 500; i–l) 30 wt% PBA*_n_*‐CTA with *X*_n_ = i) 96, j) 182, k) 332, l) 500; and m–p) 40 wt% PBA*_n_*‐CTA with *X*_n_ = m) 96, n) 182, o) 332, p) 500. Samples were printed using a fixed mass ratio of DEAm:PETA of 2:1 with 5 wt% TEOA and 0.05 wt% DCPI. The scale bars are 60 nm.

To further investigate the nanostructure of the material surfaces, AFM was performed. **Figure** **5** shows modulus maps of printed objects determined by AFM, revealing distinct nanostructures characterized by localized soft (darker areas) and hard (lighter areas) domains. The localized soft and hard domains are also evident in lower‐magnification images (Figure S19, Supporting Information). Overall, the nanostructure could be systematically tuned by varying the loading and chain length (*X*_n_) of PBA*_n_*‐CTA.

For materials with a fixed PBA*_n_*‐CTA *X*_n_ of 96 and increasing PBA*_n_*‐CTA loadings, isolated spherical domains were observed at low loadings of 10 or 20 wt% (Figure 5a,e). Increasing the content to 30 wt% resulted in a transition to elongated domains (Figure 5i), while at 40 wt%, a bicontinuous structure emerged (Figure 5m). A similar evolution from globular, to elongated, and then to bicontinuous morphologies was observed upon increasing the PBA*_n_*‐CTA content for sample sets containing higher *X*_n_ PBA*_n_*‐CTA (Figure 5; Table S11, Supporting Information). Additionally, increasing the chain length (*X*_n_) while maintaining a fixed PBA*_n_*‐CTA loading generally resulted in more connected morphologies. This trend was particularly evident in samples with 30 wt% PBA*_n_*‐CTA, where the materials exhibited an elongated morphology at *X*_n_ = 96 and a fully bicontinuous structure at *X*_n_ = 500.

In a typical diblock copolymer system where microphase separation occurs, the morphology primarily depends on the volume fraction (*f*) of each block and the segregation strength between the two blocks (*χN*).^[^ ^38^ ^]^ For the PIMS process, the resulting morphology arises from a competition between microphase separation and the kinetic arrest caused by in situ crosslinking and gelation.^[^ ^36^, ^38^ ^]^ In this work, a full morphological evolution was observed from globular‐, to elongated‐, and then to bicontinuous morphologies, by increasing the loading, analogous to *f*, of PBA*_n_*‐CTA from 10 to 40 wt% at the same *X*_n_ (Table S11, Supporting Information). *χ* was estimated to be 0.1825 at 25 °C for PBA‐*b*‐P(DEAm‐*stat*‐PETA), with a positive *χ* indicating net repulsion between the two blocks.^[^ ^39^ ^]^ By maintaining a constant PBA*_n_*‐CTA loading and a fixed mass ratio of DEAm to PETA, increasing *X*_n_ of PBA*_n_*‐CTA from 96 to 500 led to a larger *N*_total_ (Table S9, Supporting Information). Consequently, the higher χ*N* contributed to a more interconnected morphology, as observed in Figure 5i–l for 30 wt% of PBA*_n_*‐CTA. In both cases, it is the variation of either *f* or *χN* that drives microphase separation and dominates the morphological transition, with negligible impact from variation in the gel point.

In a final analysis of the material nanostructure, the PBA*_n_*‐CTA domain width (*D*_PBA_) and domain spacing (*d*_AFM_) obtained from AFM were analyzed, where *d*_AFM_ is defined as the center‐to‐center distance between two adjacent PBA*_n_*‐CTA domains. The *d*_AFM_ can be considered as a sum of PBA domain size and *net*‐P(DEAm‐*stat*‐PETA) domain size (Figure S20, Supporting Information). For each series of samples with a fixed PBA*_n_*‐CTA loading, both *D*_PBA_ and *d*_AFM_ increased monotonically as *X*_n_ increased from 96 to 500 (Figures S21–S25, Supporting Information). Across all samples, *D*_PBA_ and *d*_AFM_ ranged from 12 to 38 nm and from 19 to 71 nm, respectively, which aligned well with SAXS results (Table S11, Supporting Information). Furthermore, when fixing the PBA*_n_*‐CTA *X*_n_ and increasing the PBA*_n_*‐CTA loading from 10 to 40 wt%, *d*_AFM_ decreased, while *D*_PBA_ remained relatively constant (Table S11, Supporting Information). These trends show the inherent correlation between the average block copolymer size prior to kinetic arrest (*N_t_*_otal_) and the chain length (*X*_n_) of PBA*_n_*‐CTA (Table S9, Supporting Information).

To assess the potential for spatial variability in nanostructure due to light absorption and scattering gradients, we investigated the domain spacing and morphology of PIMS materials at different points along the two optical pathways in xolography: along the 405 nm light sheet (*x*‐direction) and in the direction of red‐light propagation (*z*‐direction). Two prisms with varying thicknesses (8 × 10 × 0.5 mm and 8 × 10 × 1.2 mm) were printed and post‐cured under identical conditions, and the middle and edge regions of each prism, representing distinct points along the 405 nm light pathway, were analyzed (Figure S26, Supporting Information). Additionally, the variation in prism thickness allowed us to examine potential anisotropy in nanoscale morphologies arising from red‐light attenuation in the *z*‐direction. SAXS analysis revealed consistent domain spacing (*d*_SAXS_ = 38 nm) across all measured regions, regardless of cuboid thickness or position (Figure S26, Supporting Information). This consistency was further corroborated by AFM, which showed bicontinuous nanoscale morphologies and comparable domain spacing (*d*_AFM_) in both middle and edge regions (Figure S27, Supporting Information). These results confirm that PIMS materials fabricated via xolography exhibit isotropic nanostructures across macroscopic dimensions, indicating that formulation, rather than printing conditions or spatial location, dictates nanostructure formation.^[^ ^21^, ^32^ ^]^

### 2.5 Macroscale Fabrication of PIMS and PIPS Materials

The small domain spacings of the PIMS materials prepared in this work ensured optical transparency along the laser light‐sheet direction (405 nm), allowing for well‐defined objects. In contrast, materials with domain spacings exceeding 200 nm are typically formed via polymerization‐induced phase separation (PIPS).^[^ ^40^ ^]^ To compare the microphase‐separated (PIMS) and macrophase‐separated (PIPS) systems and evaluate their suitability for xolography, we synthesized an inert PBA_322_ by removing the RAFT end‐group of PBA_332_‐CTA via aminolysis (Figures S28 and S29, Supporting Information).^[^ ^41^ ^]^ A resin was then formulated using the M332‐40 recipe, albeit with PBA_332_ instead of PBA_332_‐CTA, which is named P332‐40. Both M332‐40 and P332‐40 resins were prepared and printed into rectangular prisms under the same xolography conditions. The conversion of vinyl bonds in the PIMS and PIPS samples was measured before and after postcuring (Table S12, Supporting Information). Notably, the PIPS sample showed a relatively low conversion after printing (14%), which was mainly ascribed to the increased opacity of the printed material during xolography, severely hindering the penetration of 405 nm light. This was further confirmed by examining the PIMS and PIPS materials via ultraviolet‐visible light spectrometry, showing a transmittance of 21.84% and 0.04% at 405 nm, respectively (Figure S30, Supporting Information). In addition, materials printed from all PIMS formulations were investigated via UV–Vis spectra, which exhibited a strong absorbance at 500 nm (Figure S31, Supporting Information). As investigated before (Figure S8, Supporting Information), Only two constituents, PBA‐CTA and DCPI, are photo‐sensitive in the UV–Vis range, where PBA‐CTA showed a very weak absorbance of 0.02 at 500 nm for 10 mm optical path length. Therefore, the significant light absorbance below 570 nm is likely attributable to the presence of DCPI side‐products such as the benzopinacol coupling product,^[^ ^42^ ^]^ rather than the RAFT end group of PBA‐CTA.

After post‐curing, both the PIPS and PIMS materials exhibited high vinyl bond conversion (≈90%, Table S12, Supporting Information), allowing for a comparison of their mechanical properties. As such, the hardness of these two materials was measured using a Vickers hardness test, with the PIMS samples showing a higher Vickers hardness (HV, 8.72 N mm^−2^), 29% greater than that of the PIPS object (6.76 N mm^−2^, Table S13, Supporting Information). Furthermore, the indent made on the surface of PIMS materials was well‐defined with a sharp periphery. In contrast, a rough indent with conspicuous chipping and spalling^[^ ^43^ ^]^ was observed on the PIPS object, which was mainly ascribed to its relatively high brittleness and the anisotropic properties due to the coarser structures (Figure S32, Supporting Information).^[^ ^41^ ^]^ In fact, the improvement of hardness and toughness for PIMS materials with globally disordered bicontinuous morphologies can be attributed to the large interfacial area between soft and hard nanodomains. This facilitates an efficient stress transfer process, and thus a relatively uniform stress distribution applied to the materials.^[^ ^44^ ^]^

After the optimization of printing parameters (Figure S33, Supporting Information), both the PIMS and PIPS materials exhibited good resolution on the front facet of a ball‐in‐cage model directly after 3D printing (Figure S34a,e, Supporting Information). However, in the PIPS system, increased opacity due to the macrophase separation led to significant light attenuation along the 405 nm optical path. This resulted in the absence of curing in the center of the cuvette and thus the absence of the centrally located ball, as well as noticeable distortions in the side facets of the cage (Figure S34g, Supporting Information) and the central region of the material (Figure S34f,h, Supporting Information). While reducing the energy dose mitigated over‐curing on the side, it also reduced the conversion, leading to the collapse of the cage (Figure S34f–h, Supporting Information). Due to the low conversion in the PIPS system, the material lacked sufficient rigidity to withstand the washing and post‐curing process, resulting in permanent deformation and deviation from the intended geometry. In contrast, the PIMS material maintained a consistent structure without noticeable deformation after post‐treatment. This highlights the superior printability of PIMS materials in xolography, attributed to their unique nanostructures, which provide both adequate transparency and rigidity.

### 2.6 Xolography for Auxiliary‐Free 3D Printed PIMS Materials

**Figure 6:**
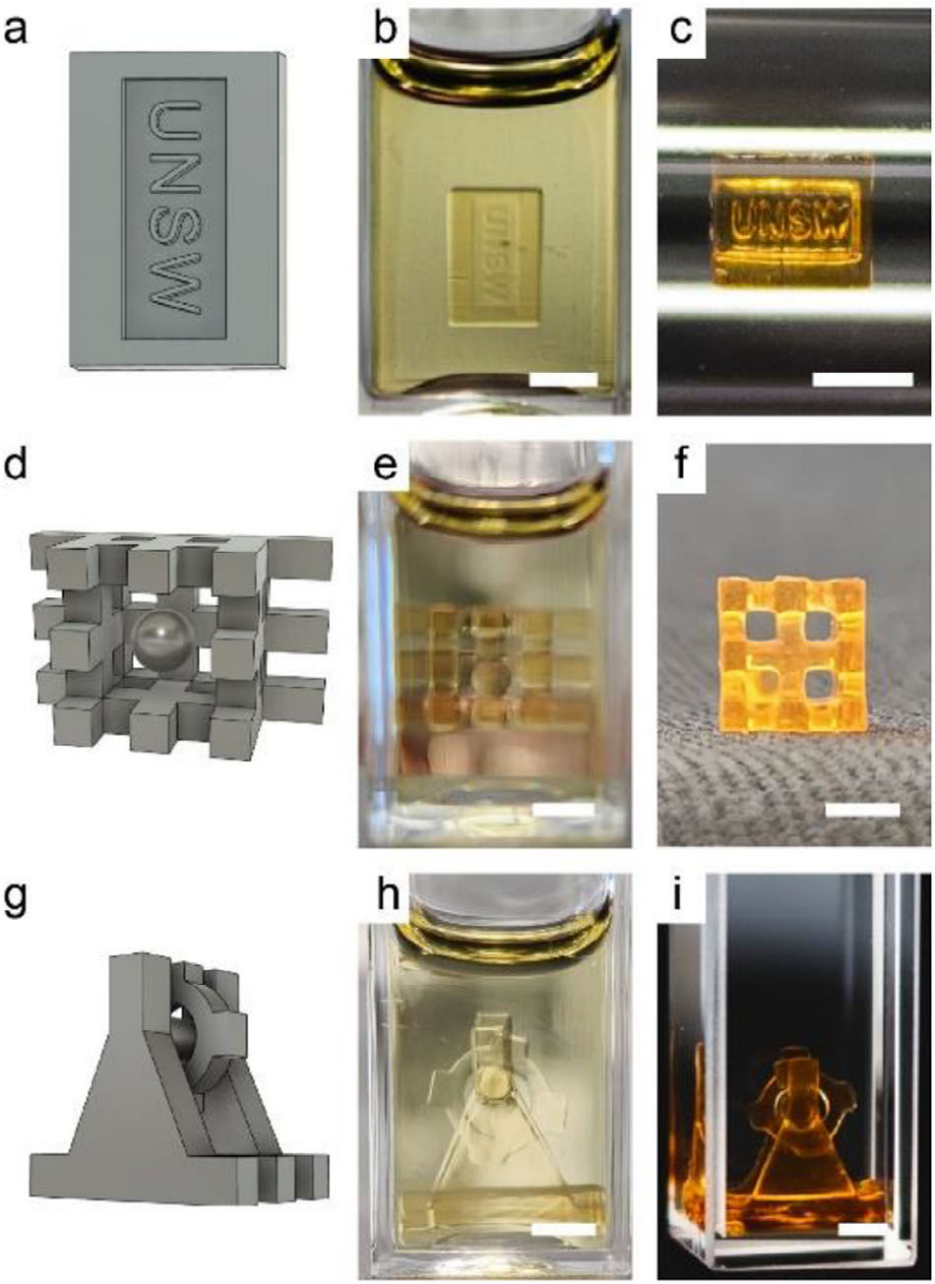
Photographs of fabricated auxiliary‐free objects. a,d,g) 3D models and printed objects b,e,h) before and c,f,i) after postprocessing. Three objects were printed at (a) 1.2 mm min^−1^ and d,g) 2 mm min^−1^ from the M500‐40 formulation. The scale bars are 3 mm.

As noted previously, the ability to manufacture auxiliary‐free structures via xolography provides significantly increased geometrical freedom during 3D printing. To successfully prepare auxiliary‐free materials, it requires two key conditions: high‐viscosity resins and rapid printing speeds. These factors work synergistically to prevent object sedimentation during the xolography process, enabling the creation of intricate, multicomponent materials in a single fabrication step. To demonstrate this capability, we selected the M500‐40 resin, the highest viscosity used in this study (1034 mPa s, Table S8, Supporting Information). First, we designed an unsupported test piece featuring the letters “UNSW” written at its center (**Figure** **6**a). Printed at 1.2 mm min⁻^1^ with an intensity of 4.8 mW mm^−2^, the structure remained upright in the uncured resin  after print (Figure 6b), with all letters clearly visible before and after postprocessing (Figure 6c). Subsequently, we designed a more complex model: a fixed cage containing a free‐floating 2 mm‐diameter ball (Figure 6d). Immediately after printing, the ball remained suspended before settling on the cage floor (Figure 6e). Postprocessing preserved its mobility (washing and postcuring), allowing free movement within the cage (Figure 6f).

To further demonstrate the capabilities of this approach, a functional waterwheel model was designed and printed (Figure 6g). This design featured an unsupported wheel situated around an axle, which was itself fixed to the base of the waterwheel. Once again, one‐step printing via xolography yielded a well‐defined, multicomponent object (Figure 6h,i). Importantly, the wheel exhibited free rotation around the axle, as clearly demonstrated in the accompanying video (Video S1, Supporting Information). This video showcases the rapid rotation of the wheel, powered by a stream of water from a syringe. These examples collectively demonstrate that auxiliary‐free printing is achievable using PIMS resin (M500‐40), paving the way for the fabrication of even more intricate and functional 3D‐printed structures in the future.

## 3 Conclusion

This study successfully demonstrated the ability to tune the structure of 3D‐printed materials across multiple length scales using PIMS resins in xolography, achieving precise control from the nanoscale to the macroscale. PIMS resins were systematically formulated and optimized, by considering key factors such as transparency, homogeneity, chemical reactivity, and the segregation strength that drives microphase separation. Through systematic exploration and refinement of printing parameters, materials with the smallest positive (80 µm) and negative (80 µm) features were attainable. More viscous resins provided more accurate replication of digital models due to their higher reactivity and tendency to restrict sediment during the xolography process. Investigation of the 3D printed material nanostructures revealed tunable domain spacings ranging from 18 to 66 nm, as observed via SAXS analysis. AFM further demonstrated morphological transitions from globular to elongated and eventually to bicontinuous nano‐domains as the PBA*_n_*‐CTA chain length or loading increased. Notably, the domain spacings followed a predictable power law dependence on the PBA*_n_*‐CTA chain length, offering a straightforward method for nanostructure tuning.

Following optimization, high‐viscosity resins were successfully used to fabricate auxiliary‐free objects via xolography, a task which is difficult or impossible to conduct via conventional layer‐by‐layer 3D printing techniques. Free‐floating structures, including a ball‐in‐cage model, were printed with unconstrained mobility via xolography. Furthermore, a fully functional waterwheel powered by a stream of water was successfully fabricated in a single pass, demonstrating the ability to print interlocking and unconstrained components in one step. In conclusion, the PIMS xolography system developed in this study holds significant promise for fabricating complex, unconstrained, and free‐floating materials for biomedical and other applications. To realize the full potential of PIMS in xolography, future work will also focus on greener synthesis and the production of recyclable materials, in line with current sustainability goals. Altogether, this work highlights the versatility and power of combining PIMS resin technology with the volumetric printing capabilities of xolography.

## Conflict of Interest

The authors declare no conflict of interest.

## Supporting information



Supporting Information

Supplemental Video 1

## Data Availability

The data that support the findings of this study are available from the corresponding author upon reasonable request.
