# Supplementary material for: Xolography for Rapid Volumetric Production of Objects from the Nanoscopic to Macroscopic Length Scales
Source: Adv Mater. 2025 Jun 29;37(37):2503245. doi: 10.1002/adma.202503245 (PMC12447021; doi:10.1002/adma.202503245)
Supplement: Supplementary file 1 — Supporting Information [file ADMA-37-2503245-s002.pdf]

ADVANCED MATERIALS

Supporting Information

for *Adv. Mater.*, DOI 10.1002/adma.202503245

Xolography for Rapid Volumetric Production of Objects from the Nanoscopic to Macroscopic Length Scales

Xichuan Li, Yuan Xiu, Kenny Lee, Jin Zhang, Nathaniel Corrigan* and Cyrille Boyer**

Supporting Information

Xolography for Rapid Volumetric Production of Objects from the Nanoscopic to Macroscopic Length Scales

Xichuan Li,[1] Yuan Xiu,[1] Kenny Lee,[1] Jin Zhang,[2*] Nathaniel Corrigan[1,3*] and Cyrille Boyer[1,3*]

[1]Cluster for Advanced Macromolecular Design, School of Chemical Engineering, University of New South Wales, Sydney, NSW 2052, Australia

[2]School of Mechanical and Manufacturing Engineering, University of New South Wales, Sydney, NSW 2052, Australia

[3]Australian Centre for Nanomedicine, University of New South Wales, Sydney, NSW 2052, Australia

*Email: jin.zhang6@unsw.edu.au, n.corrigan@unsw.edu.au, cboyer@unsw.edu.au

Materials

Unless otherwise stated, all materials were used as received. *n*-butyl acrylate (BA, ≥99%), pentaerythritol tetraacrylate (PETA, tetraacrylate by GC 60.0-90.0%, triacrylate by GC 10.0-40.0%), tributylamine (≥98.5%), *N*-methyldiethanolamine (≥99%), hexylamine (99%), methyl acrylate (99%), 2,2'-azobis(2-methylpropionitrile) solution in acetone (AIBN solution, 12 wt%), and polymethacrylate cuvettes (≥ 3 mL, 10 mm path length, optical grade polymethacrylate) were purchased from Sigma-Aldrich. *N,N*-Diethylacrylamide (DEAm, 98.0%), triethanolamine (TEOA, 98%), acetonitrile (HPLC grade), diethyl ether anhydrous (AR grade), methanol (AR grade), and magnesium sulphate (anhydrous powder) were purchased from Chem-Supply. 2-(Butylthiocarbonothioylthio) propanoic acid (BTPA) was purchased from Boron Molecular. Chloroform-D ($CDCl_3$, 99.8%) was purchased from Cambridge Isotope Laboratories. Dimethylacetamide (DMAc, HPLC grade) was purchased from RCI Labscan limited. The dual-color photoinitiator (DCPI) was provided by xolo GmbH (424 g mol^{-1} as provided by xolo GmbH).

Methods

Synthesis of PBA_n-CTA

Typical protocol for the synthesis of PBA_{96}-CTA: AIBN powder was obtained by drying the AIBN solution with nitrogen gas. *n*-Butyl acrylate (85.00 g, 0.6632 mol), BTPA RAFT agent (1.5810 g, 0.0066 mol) and AIBN (0.1634 g, 0.0010 mol) were dissolved in acetonitrile (172.58 g, 4.2041 mol). The resin mixture containing a stirring bar was deoxygenated under nitrogen for 60 min before being put in oil bath for 18 h at 60 °C. The polymerization was terminated by cooling down in ice bath for 30 min and then exposing to air. The yellow crude product was obtained via rotary evaporation to remove volatile components, followed by a precipitation in cold methanol (10 times volume of crude PBA_n-CTA) for two times. After another rotary evaporation, the yielded product was transferred to the vacuum oven at 40 °C to remove volatile residuals. Other PBA_n-CTAs were synthesized by using the same protocol.

Aminolysis of PBA_{332}-CTA

PBA_{332}-CTA (50.0 g, 1.2 $\times 10^{-3}$ mol) was dissolved in acetonitrile (170 mL). The solution was deoxygenated by purging with dry nitrogen gas for 90 min. To this solution, methyl acrylate (0.6 mL, 6.6 $\times 10^{-3}$ mol) was injected via a syringe, followed by the injection of hexylamine (0.6 mL, 4.6×10^{-3} mol). The reaction was allowed to react for 15 h at 22°C. After 15 h, the yellow color of the RAFT end group was still present, so more methyl acrylate (0.2 mL, 2.2×10^{-3} mol) and hexylamine (0.6 mL, 4.6×10^{-3} mol) were injected to the reaction solution. The reaction was allowed to react for another 5 h. The reaction solution was then concentrated by rotary evaporation, then redissolved in chloroform and washed five times with Milli-Q water (5 × 60 mL). The polymer solution was then dried over anhydrous magnesium sulphate ($MgSO_4$), filtered and

reduced in volume by rotary evaporation. The resulting mixture was used without further purification. GPC (RI, DMAc): M_n = 39.9 kg mol^{-1}, $Đ$ = 1.18.

Resin preparation

Resins were prepared using the same procedure, which is illustrated in a representative example here. Specifically, for the preparation of resin mixture (10.5 g) consisting of 40.00 wt% PBA_{500}-CTA, 36.67 wt% DEAm, 18.33 wt% PETA, 5.00 wt% TEOA and 0.05 wt% DCPI: PBA_{500}-CTA (4200 mg, 0.065 mmol) was added to a 20 mL glass vial wrapped with foil, followed by the addition of DEAm (3750 mg, 29.486 mmol) capable of dissolving PBA_{500}-CTA via repeated mechanical stirring, sonication and vortexing. Afterwards, PETA (1925 mg, 5.463 mmol) and TEOA (525 mg, 3.519 mmol) were added to the mixture, which was then put on the vortex for further mixing. The stock solution of DCPI (5 wt% in DEAm) was prepared and 105.00 mg of this solution was added, then the whole mixture was put on rotating rollers to make it completely homogeneous. The resin mixture was stored in a dark environment before being distributed to polymethacrylate cuvettes, which were cleaned by purging with dry nitrogen gas. These cuvettes were sealed with parafilm and centrifuged at 3000 rpm for 1 min at 24 °C to remove air bubbles and were kept in a box prior to being printed. Preparation of other resin systems followed the same protocol.

Model design

All models were designed in Fusion 360 and converted to stereolithography (.stl) files, which were transferred to the volumetric 3D printer (Xube). The inbuilt software in the Xube was utilized to further convert .stl files to the video files available for printing. All parameters were set on Xube (print position, print speed and 405 nm light-sheet intensity) before printing. All models were printed using a single printing condition except for the model consisting of a ball-in-cage model which was printed with three conditions in a consecutive process as follows.

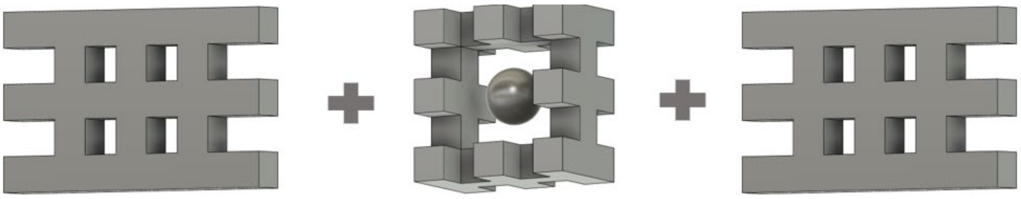

Printing and post-processing

Printing was performed on Xube with a 405 nm laser light-sheet (2 × 350 mW diode lasers) and a 3840 × 2160 pixel (UHD) digital light projector. The projector's mercury lamp produced light in the 540-720 nm spectral region (referred to as red light). The maximum build size was 10 × 17.8 × 10 mm (x, y, z). After the resin was prepared in the vat (polymethacrylate cuvette), it was placed in the vat holder and the corresponding model file (xolo) was selected. The print parameters were set within the software and the xolography 3D printing

was performed to acquire the targeted objects. SAXS and AFM samples were printed using a rectangle prism model (6 × 8 mm) with various thicknesses of 0.8 mm and 2 mm, respectively.

After print, the vat was removed from the Xube and the uncured liquid resin was removed via squash pipette. Then the vat was refilled with diethyl ether and rinsed several times through repeated squash pipetting. Sonication (~15 s) was conducted for viscous resin systems in the same cuvette filled with clean solvent to further remove viscous resin from the surface of the printed sample. After confirming all residual resin was removed from the cuvette and the printed object, it was left in the fume hood, allowing for air dry under ambient conditions for 10 min. Unless other stated, all dried objects in the cuvette were post-cured under the irradiation of violet light (λ_{max} = 405 nm, 166 mW cm^{-2}) for 5 min.

The emission spectrum of the digital light projector

An Owave VIS-Compact USB spectrometer (Qwave Spectrometer AFBR-S20W2VI) with high resolution and sensitivity was used to measure the emission spectrum of the projected light.

Measurement of 405 nm light-sheet

The 405 nm light-sheet was characterized with a camera sensor (XMX264, 2/3" format, 8.8 mm × 6.6 mm) on both sides of the cuvette (print vat) center over a ± 10 mm range with a step size of 0.2 mm. The exposure time was set to 10 ms to ensure adequate signal strength for image acquisition. The recorded images were analyzed to determine light sheet thickness by counting pixels and converting them using the pixel size.

Nuclear magnetic resonance (NMR)

All NMR spectra were recorded on Bruker Avance III 400 MHz spectrometer and all chemical shifts are reported in ppm (*δ*) relative to tetramethylsilane (TMS), referenced to the chemical shifts of residual solvent resonances. All ^1H NMR samples were prepared in $CDCl_3$.

Gel permeation chromatography (GPC)

Gel permeation chromatography (GPC) was employed to determine the molecular weight and dispersity of the synthesized polymers. The GPC system consists of DMAc eluent (containing 0.03% w/v LiBr and 0.05% w/v 2,6-dibutyl-4-methylphenol (BHT)) at 50 °C (flow rate of 1 mL min^{-1}) with a Shimadzu modular system comprising a SIL-10AD auto-injector, a Polymer Laboratories 5.0 μm bead-size guard column (50 × 7.5 mm^2) followed by three linear PL (Styragel) columns (10^5 Å, 10^4 Å, 10^3 Å) and an RID-10A differential refractive-index (RI) detector. The equipment was calibrated based on commercial poly (methyl methacrylate) (PMMA) standards with molecular weight of 200-10^6 g mol^{-1}.

Fourier transform near-infrared (FTNIR) spectroscopy

FTNIR spectroscopy was performed using a Bruker Vertex 70 Fourier transform spectrometer. FTNIR spectroscopy was used to measure vinyl bond conversion of solid samples by comparing the integral of the C-H vinylic stretching overtone at 6115-6230 cm^{-1} between solid printed samples and a sample of uncured resin. Uncured resin (3 mL) was placed in a 1 × 1 cm quartz cuvette, and an absorption spectrum was obtained by scanning from 4000-12000 cm^{-1}. A solid 3D printed sample was measured for its thickness using a digital caliper, then placed in the cuvette and another absorption spectrum was scanned. The final conversion was calculated using **Equation S1 (Supporting Information)**:

$$\text{Conversion (\%)} = 100 \times \left(1 - \left(\frac{int_s}{int_0} \times \frac{t_{\text{cuvette}}}{t_{\text{sample}}}\right)\right) \quad \textbf{(S1)}$$

where int_s indicates the integral of the peak from 6115-6230 cm^{-1} for the printed object, int_0 is the integral of the peak from for the unpolymerized resin; t_{sample} is the thickness of the printed sample, and t_{cuvette} is the path length of liquid resin in the cuvette. Integrals were calculated using OPUS software 7.5.

Ultraviolet-Visible light (UV-Vis) spectroscopy

All UV-Vis spectra were recorded with a Varian Cary 300 spectrophotometer. Unless otherwise stated, scans were conducted in the range of 800-200 nm at 600 nm min^{-1}. For measurement of the absorbance vs wavelength profiles, samples were placed in a quartz cuvette while the instrument was zeroed at 800 nm. To acquire the molar extinction coefficient of DCPI, a series of solutions (DCPI in acetonitrile) were prepared with gradient concentrations, ranging from 2 mg mL^{-1} to 5.5 mg mL^{-1}. At least eight points were measured to construct a calibration curve of concentration vs absorbance, where the absorbance was constrained between 0.2-1.4. The absorbance at 405 nm (corresponding to laser light-sheet wavelength) was utilized to conduct calibration for the molar extinction coefficient. Similarly, a variety of BTPA solutions ranging from 2 mg mL^{-1} to 9 mg mL^{-1} were prepared and the absorbance at 430 nm (n→π* transition of the thiocarbonylthio group) was used for calibration.[1] The molar extinction coefficient was calculated based on **Equation S2 (Supporting Information)**.

$$A = \varepsilon \cdot c \cdot l \quad \textbf{(S2)}$$

where A is the absorbance, ε (L mol^{-1} cm^{-1}) is the molar extinction coefficient, c (mol L^{-1}) is the concentration of the tested sample, l (cm) is the optical path length.

For measurement of the absorbance spectrum of the metastable merocyanine form of DCPI in resin mixture, a specific formulation was selected, consisting of 42.11 wt% PBA_{500}-CTA, 38.60 wt% DEAm, 19.30 wt% PETA and 0.05 wt% DCPI, but without TEOA to avoid polymerization. This recipe is similar to the one with 40 wt% PBA_{500}-CTA (M500-40) with TEOA. Then, the resin was pipetted into a quartz cuvette, followed by

being irradiated with 405 nm light (Thorlabs 405 nm LED with collimation adaptor) at 76.4 mW cm^{-2} (measured by a Newport 843-R power meter) for 30 seconds, which was then put into UV-Vis spectrometer. A scan was conducted from 700 to 300 nm at a scan rate of 600 nm min^{-1} to reduce measuring time so as to offset the unwanted effect brought by the thermal reversion of DCPI. The spectra of DCPI were also recorded before and after 405 nm irradiation. Likewise, UV-Vis absorbances of DEAm, PETA, TEOA, 10 wt% PBA_{500}-CTA in DEAm and 0.05 wt% DCPI in DEAm were measured to construct light distribution profiles in the printer-Xube (referring to **light distribution estimation** section).

For determination of the half-life of DCPI, reverting to the spiropyran from the metastable merocyanine form, all resin mixtures were prepared referring to **resin preparation** section without the addition of TEOA. The resin was pipetted into a quartz cuvette before being put into the UV-Vis spectrometer. The absorbance for measurement was determined to be 585 nm (corresponding to the assumed wavelength of digital light projector) and zeroed before measurement. After recording the absorbance at 585 nm for 20 s to offer a baseline reference, the sample was removed from the UV-Vis spectrometer and irradiated with 405 nm LED light at 76.4 mW cm^{-2} for 30 s, which was then immediately put back into the UV-Vis spectrometer for subsequent recording of absorbance at 585 nm. Measurement was not stopped until the absorbance plateaued. Data obtained was exported to Microsoft Excel with a truncation to remove the points corresponding to the period when the cuvette was irradiated with 405 nm light. The data was plotted and fitted with one phase decay equation and no other constraints were selected for model fitting.

Light distribution estimation

In Xube, 405 nm laser light source is diverted into two beams with equivalent intensity which radiate the print vat from two sides. As the UV-Vis spectra showed PBA_n-CTA and DCPI absorbed violet light more significantly compared to other components (DEAm, PETA, TEOA) in the formulation used in this study (**Figure S8, Supporting Information**), only PBA_n-CTA and DCPI were the main factors affecting the violet light distribution (405 nm) in the vat containing precursors. The calculation was conducted based on Beer-Lambert law (**Equation S2, Supporting Information**).

Viscosity measurements

All samples were measured using a Brookfield DV3T viscometer equipped with a CPA-52Z spindle and temperature-controlled TC-150SD heating components. Recording was performed at 25 ± 0.2 °C. Data points were recorded every 10 s throughout the 5 min period until the viscosity values were constant. The results were invalid when the torque was out of recommended range (10-90 %), while the revolution speed (rpm) should be as high as possible to improve the accuracy of each measurement. It was noted that there was a slight deviation in the viscosity results (< 5%) for all samples except for two with very low viscosities (M96-10 and M182-10).

Scanning electron microscopy (SEM)

All samples were prepared on a stub with conductive tape and coated with platinum coating with 30 nm thickness by virtue of a Leica ACE600 sputter coater. SEM images were acquired using a field-emission NanoSEM 230 instrument equipped with a 3 kV accelerating voltage and a secondary electron detector.

Atomic force microscopy (AFM)

All AFM tests were performed on the Bruker Dimension ICON SPM, equipped with a Nanoscope V controller (software version 9.70). An OTESPA-R3 probe (from Bruker AFM probes) was utilized to conduct tapping mode measurements. Another SCANASYST probe (from Bruker AFM probes) was selected to measure mechanical properties using peak force tapping mode on the surface of the printed object. The scanning size was set to be 300 nm and 1 μm. The scanning rate was set at around 0.6-0.7 Hz with a peak force of approximate 500 pN. The feedback gain was adjusted accordingly to optimize the tracking of the specimen surface, without obvious feedback noise. The resolution of the image was set to be 512 pixels per line for 1 μm scan size and 256 pixels each line for 300 nm scan size. For peakforce QNM measurements, the tip was calibrated via thermal tuning method. AFM results were analysed using NanoScope Analysis software (version 1.7). Regarding the statistical length analysis, at least 50 points of each sample were counted manually to determine average domain spacing and domain size by means of ImageJ software. Average domain spacing (*d*) and PBA-CTA domain width (*D*) were calculated via **Equation S3 (Supporting Information)**:

$$d = \frac{\sum_{i=1}^{n} d_i}{n} \quad \textbf{(S3)}$$

where *n* is the number of statistical points, *d* was defined as the centre-to-centre distance between two adjacent PBA-CTA domains. Likewise, the average PBA-CTA domain width (*D*) was determined using the same equation.

Small-angle X-ray scattering (SAXS)

SAXS measurements were conducted on an Anton Paar SAXSPoint 2.0 system with a Cu K_α (λ = 0.154 nm) microfocus X-ray source and Dectris Eiger 1M detector. Data was acquired at room temperature, under vacuum for 5 min from the sample chamber at a sample-to-detector distance of 0.575 m. Objects were printed at the thickness of 0.8 mm. Data was reduced to 1D by radial averaging the 2D detector after converting pixel positions to $q = (4\pi/\lambda)\sin\theta$, where 2θ is the scattering angle. The domain spacing could be obtained using **Equation S4 (Supporting Information)**:

$$d_{\mathrm{SAXS}} = \frac{2\pi}{q} \quad \textbf{(S4)}$$

SAXS fitting using the Teubner-Strey (T-S) model

The sharpness and position of SAXS peaks of printed microphase-separated samples were fitted by virtue of T-S model[2] in SasView software, referring to **Equation S5 (Supporting Information)**:

$$I(q) = \frac{1}{\mathrm{a}_2 + \mathrm{c}_1 q^2 + \mathrm{c}_2 q^4} + \mathrm{b} \quad \textbf{(S5)}$$

where $q = (4\pi/\lambda)\sin\theta$, λ is the wavelength; 2θ indicates the scattering angle; b is background scattering; a_2, c_1, c_2 are fitting parameters utilized to calculate domain spacing (d_{TS}), correlation length (ξ) and the amphiphilicity factor (f_a) with following **Equations S6-S8 (Supporting Information)**:

$$d_{\mathrm{TS}} = 2\pi\left[\frac{1}{2}\left(\frac{\mathrm{a}_2}{\mathrm{c}_2}\right)^{1/2} - \frac{1}{4}\frac{\mathrm{c}_1}{\mathrm{c}_2}\right]^{-1/2} \quad \textbf{(S6)}$$

$$\xi = \left[\frac{1}{2}\left(\frac{\mathrm{a}_2}{\mathrm{c}_2}\right)^{1/2} + \frac{1}{4}\frac{\mathrm{c}_1}{\mathrm{c}_2}\right]^{-1/2} \quad \textbf{(S7)}$$

$$f_a = \frac{\mathrm{c}_1}{\sqrt{4\mathrm{a}_2\mathrm{c}_2}} \quad \textbf{(S8)}$$

Estimation of $\chi_{\mathrm{P(DEAm}\text{-}stat\text{-}\mathrm{PETA)}\text{-}b\text{-}\mathrm{PBA}}$ by group molar contribution method

For diblock copolymers consisting of one block which is a statistical copolymer of two various monomers, χ can be reasonably assumed using a binary interaction model[3] (**Equation S9, Supporting Information**):

$$\chi_{\mathrm{P(DEAm}-stat-\mathrm{PETA)}-b-\mathrm{PBA}} = (1-x)\chi_{\mathrm{PPETA-PBA}} + x\chi_{\mathrm{PDEAm-PBA}} + x(1-x)\chi_{\mathrm{PDEAm-PPETA}} \quad \textbf{(S9)}$$

where x is the weight fraction of DEAm in P(DEAm-stat-PPETA) block (x = 0.67). χ_{12} was calculated according to **Equation S10 (Supporting Information)**:

$$\chi_{12} = \frac{VN_{\mathrm{A}}}{RT}(\delta_1 - \delta_2)^2 \quad \textbf{(S10)}$$

where V is the reference volume (set to be 118 Å^3), R is the gas constant (1.986 cal mol^{-1} K^{-1}), T is temperature (set to be 298 K), N_A is the Avogadro's number (6.02 × 10^{23} mol^{-1}), δ [(cal cm^{-3})$^{1/2}$] is solubility parameter assumed based on the group molar contribution method put forward by Small[4] (**Equation S11, Supporting Information**):

$$\delta = \frac{d\sum G}{M} \quad \textbf{(S11)}$$

where d (g cm^{-3}) is density, M is monomer molecular weight, $\sum G$ is the sum of the molar attraction constants. Estimated δ values were as follows: δ_{PBA} = 9.13 cal$^{1/2}$ cm$^{-3/2}$, δ_{PDEAm} = 9.69 cal$^{1/2}$ cm$^{-3/2}$, δ_{PPETA} = 7.85 cal$^{1/2}$ cm$^{-3/2}$. Subsequently, χ parameters were calculated via **Equation S10 (Supporting Information)**: $\chi_{\mathrm{PDEAm-PBA}}$ = 0.0375, $\chi_{\mathrm{PPETA-PBA}}$ = 0.1992, $\chi_{\mathrm{PDEAm-PPETA}}$ = 0.4097, Then $\chi_{\mathrm{P(DEAm}\text{-}stat\text{-}\mathrm{DEAm)}\text{-}b\text{-}\mathrm{PBA}}$ was calculated using **Equation S9 (Supporting Information)**: $\chi_{\mathrm{P(DEAm}\text{-}stat\text{-}\mathrm{DEAm)}\text{-}b\text{-}\mathrm{PBA}}$ = 0.1825.

Vickers hardness test

Vickers hardness (HV) was measured on the surface of the designated sample by Vickers indentation (DuraScan-80, Struers) under the load of 0.1 kgf (1 kgf = 9.8 N). 3 replicated measurements were performed for each sample (n = 3).

Additional Data

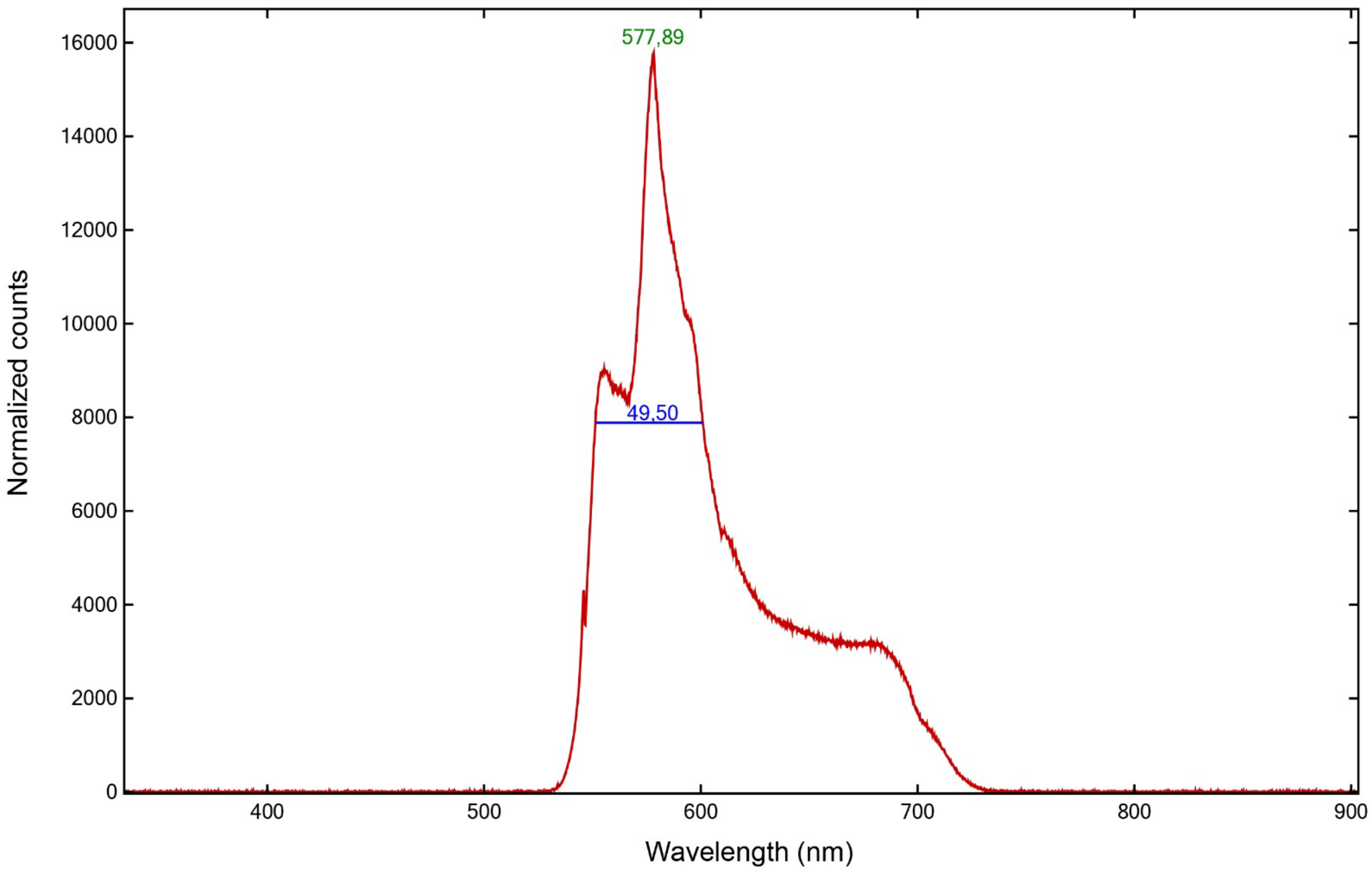

Figure S1. The emission spectrum of the projected light (referred to as red light).

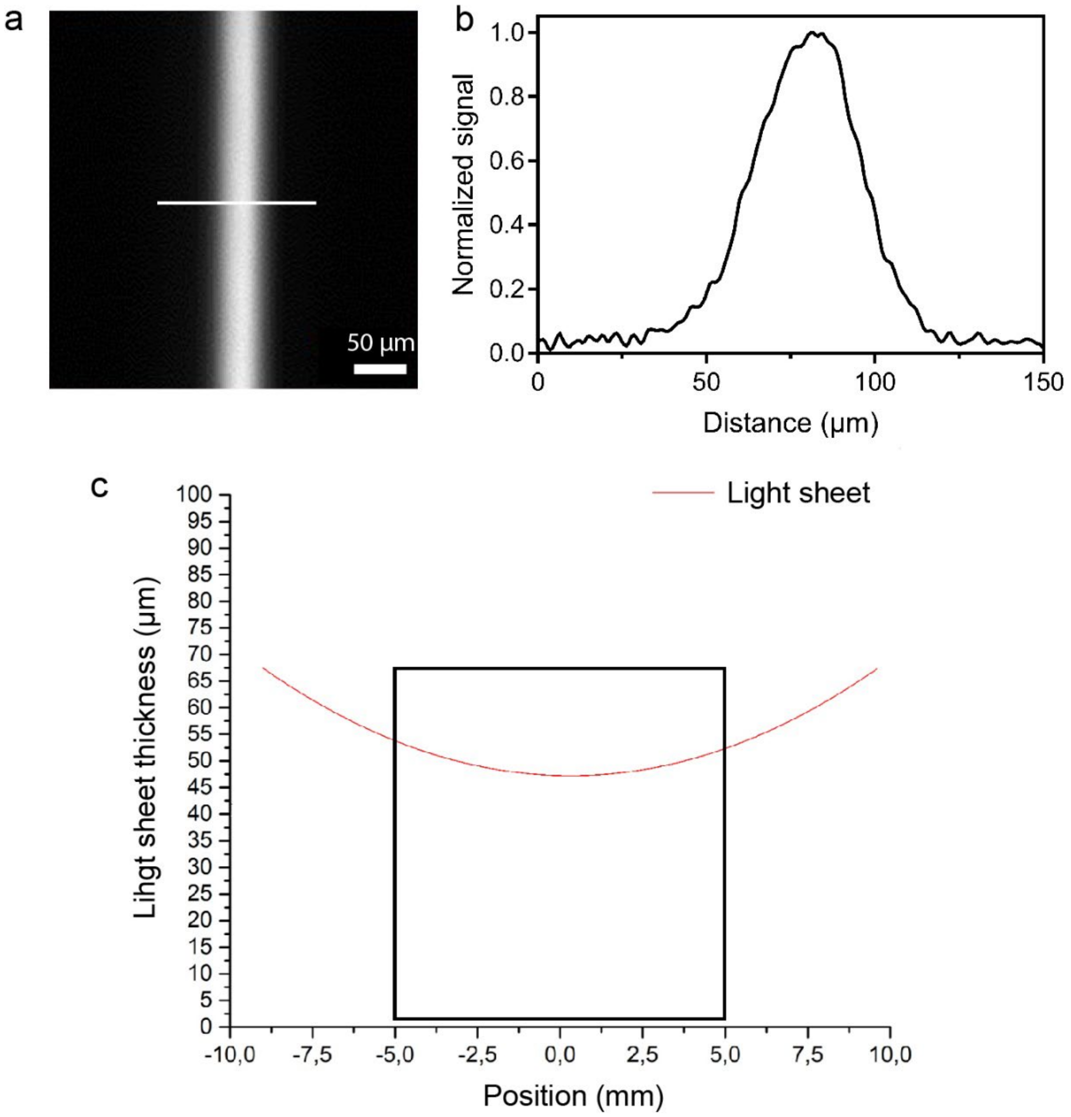

Figure S2, The optical information of 405 nm light-sheet. (a) Partial image of the light-sheet in one arm at the waist position. (b) Horizontal light intensity distribution along *z*-direction measured via ImageJ derived from the white indicator in (a). (c) The light sheet thickness was measured on both sides of the print vat (cuvette) center over a range ± 10 mm with a step size of 0.2 mm along *x*-direction. (*x*, *y*, and *z* axis are illustrated in the main text, **Figure 1a**)

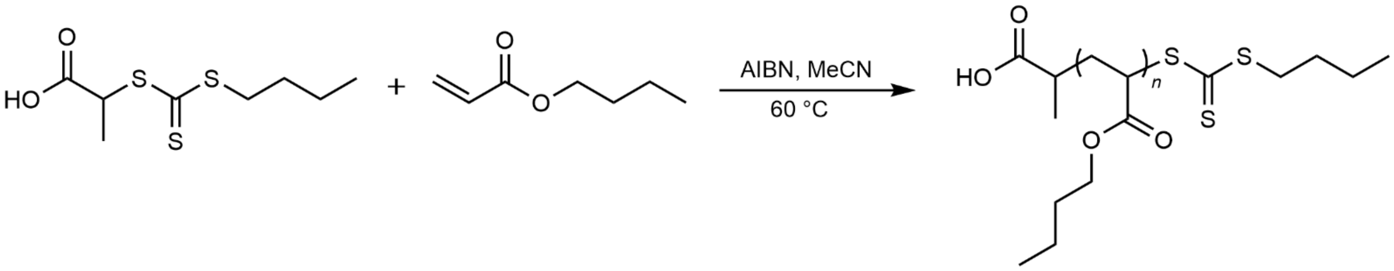

Figure S3. The general procedure of synthesizing PBA$_n$-CTAs via thermal RAFT polymerization**.** More details are available in **Synthesis of PBA$_n$-CTA**

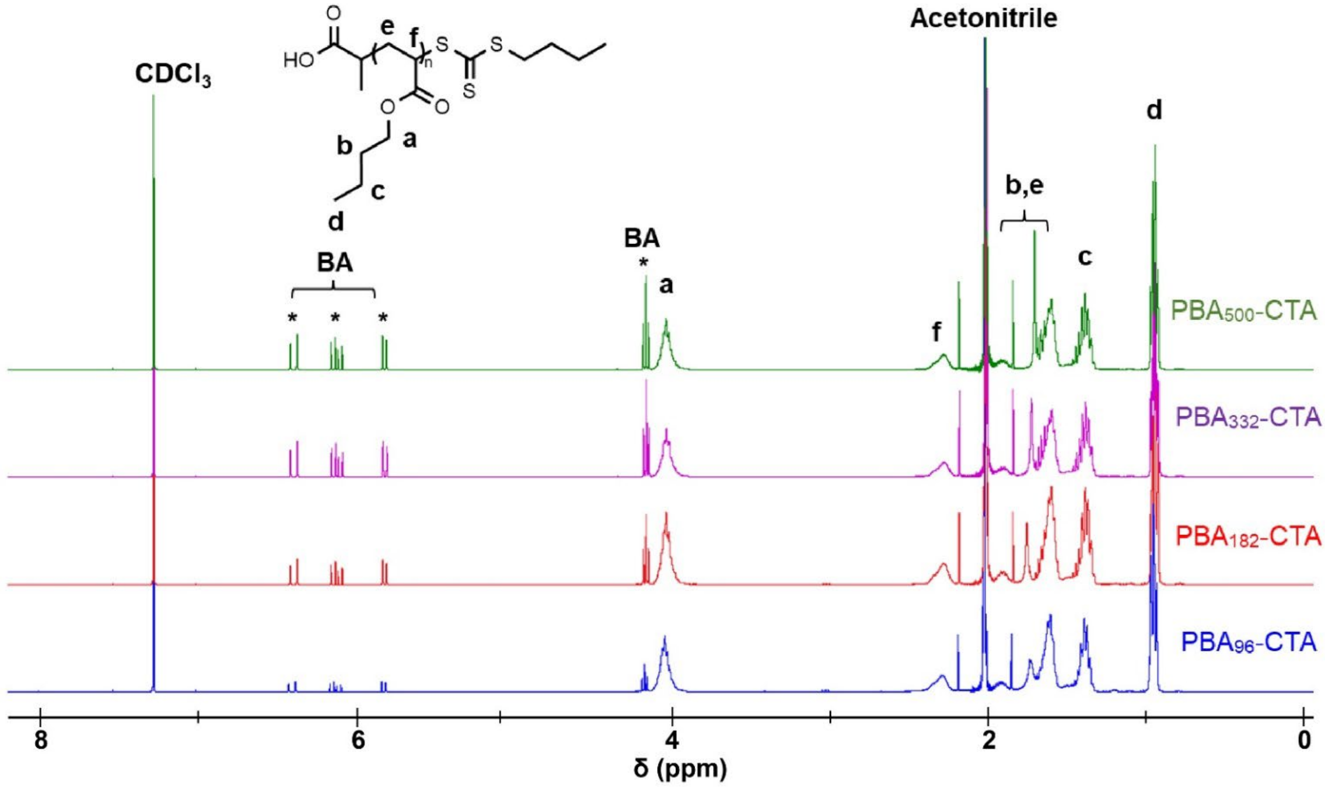

Figure S4. ^{1}H NMR spectra of crude PBA$_n$-CTAs. * -signals of protons from butyl acrylate (BA) monomer. Acetonitrile was used as a solvent for synthesis. Spectra were obtained using $CDCl_3$ as solvent recorded on Bruker Avance III 400MHz spectrometer at 298 K.

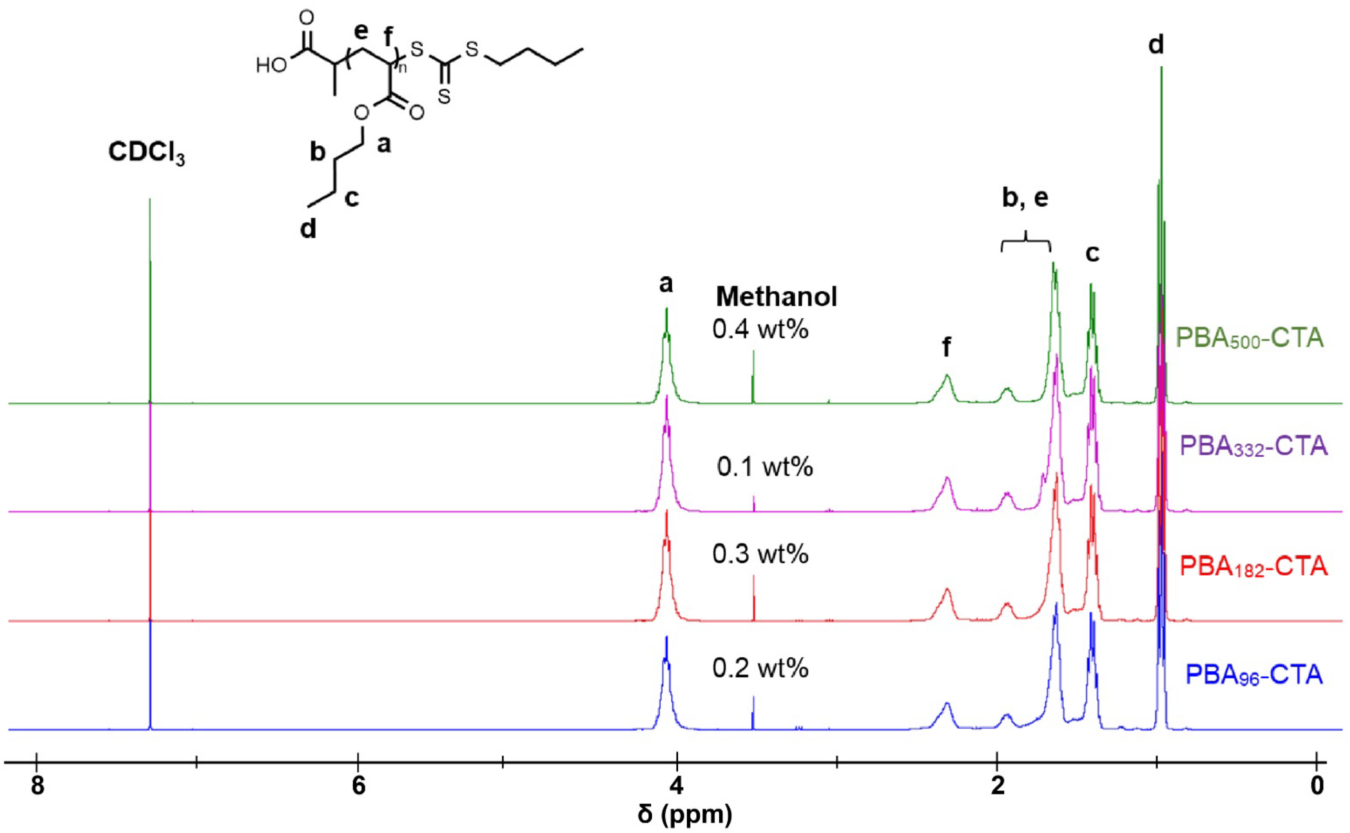

Figure S5. ^{1}H NMR spectra of purified PBA$_n$-CTAs via precipitation in methanol, rotary evaporation and vacuum drying at 40 °C. Spectra were obtained using $CDCl_3$ as solvent recorded on Bruker Avance III 400MHz spectrometer at 298 K.

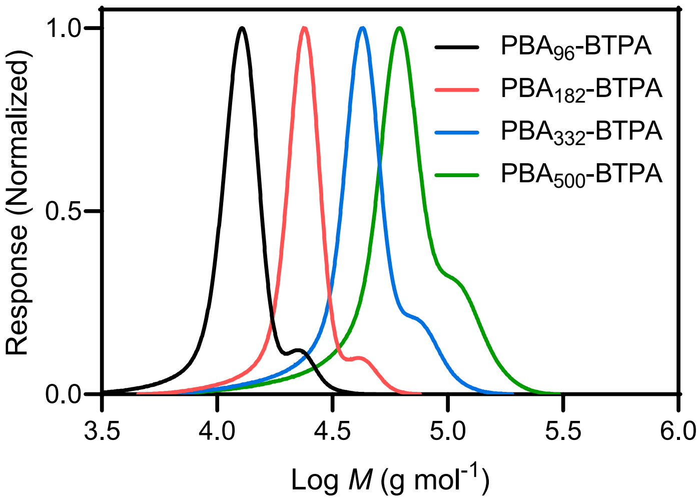

Figure S6. Molecular weight distributions of PBA_n-CTAs after purification. DMAc was used as eluent and PMMA as calibration standards.

Table S1. Characterization of PBA_n-CTAs synthesized by RAFT-mediated polymerization of *n*-butyl acrylate.

MacroCTA	Molar feed ratio [BA]/[BTPA]	^{1}H NMR[a]			GPC (RI, DMAc)[b]	
		Conversion (%)	X_n	M_n (kg/mol)	M_n (kg/mol)	*Đ*
**PBA_{96}-CTA**	100	96	96	12.5	12.4	1.10
**PBA_{182}CTA**	200	91	182	23.6	22.7	1.09
**PBA_{332}-CTA**	400	83	332	42.8	38.8	1.17
**PBA_{500}-CTA**	590	85	500	64.3	54.6	1.25

[a]-Monomer conversion was calculated based on ^{1}H NMR spectra by comparing integrals of polymers (4.06) and residual vinyl bonds from monomer (5.78 - 6.46 ppm); X_n = ([BA]/[BTPA]) × conversion; $M_n = X_n \times$ MW(BA) + MW (BTPA). Where [BA]/[BTPA] represents the molar feed ratio of BA to BTPA molecules; X_n is the calculated degree of polymerization from ^{1}H NMR, MW is molecular weight of each molecule; M_n is the calculated number-average molecular weight. [b]-DMAc (containing 0.03% w/v LiBr and 0.05% w/v 2,6-dibutyl-4-methylphenol (BHT)) was utilized as eluent with PMMA as the calibration standard.

Table S2. Monomer conversion of printed objects using various coinitiators on Xube.

Coinitiator	Tributylamine[b]	*N*-methyldiethanolamine[b]	Triethanolamine[c]
**Conversion (%)**[a]	22	41	41

[a]-Vinyl bonds from uncured resins and printed objects were determined by FTNIR to calculate monomer conversion of the printed object without post-curing (**Equation S1, Supporting Information**). [b]-The sample was printed under 0.4 mm min^{-1} and 6.4 mW mm^{-2}. [c]-The sample was printed under 0.6 mm min^{-1} and 6.4 mW mm^{-2}.

Notes: 5 wt % of various coinitiators were used while keeping other constituents the same: 20 wt% PBA_{182}-CTA, 30 wt% DEAm, 45 wt% PETA, 0.05 wt% DCPI. Under the irradiation of lower energy dose, the sample printed with triethanolamine showed the same resin conversion for gelation as the one with *N*-methyldiethanolamine. Hence, triethanolamine was chosen for subsequent experiments.

Table S3. Number-average molecular weights and dispersity of polymers via chain extension of PBA_{182}-CTA with DEAm using various concentrations of coinitiator and photoinitiator on Xube.

Resin	$m_{(PBA\text{-}CTA)}$[a]	$m_{(DEAm)}$[a]	$m_{(TOEA)}$[a]	$m_{(DCPI)}$[a]	M_n (kg mol^{-1})[b]	*Đ*
**1**	20	30	1		23.9	1.10
**2**	20	30	5	0.01	27.5	1.16
**3**	20	30	10		29.3	1.19
**4**	20	30	1		23.6	1.09
**5**	20	30	5	0.05	29.0	1.19
**6**	20	30	10		31.2	1.24
**7**	20	30	1		23.9	1.09
**8**	20	30	5	0.10	29.1	1.15
**9**	20	30	10		31.2	1.27

[a]-*m* refers to the weight ratio in the resin. [b]-The number-average molecular weight of linear PBA_{182}-*b*-PDEAm after print (0.4 mm min^{-1} and 6.4 mW mm^{-2}).

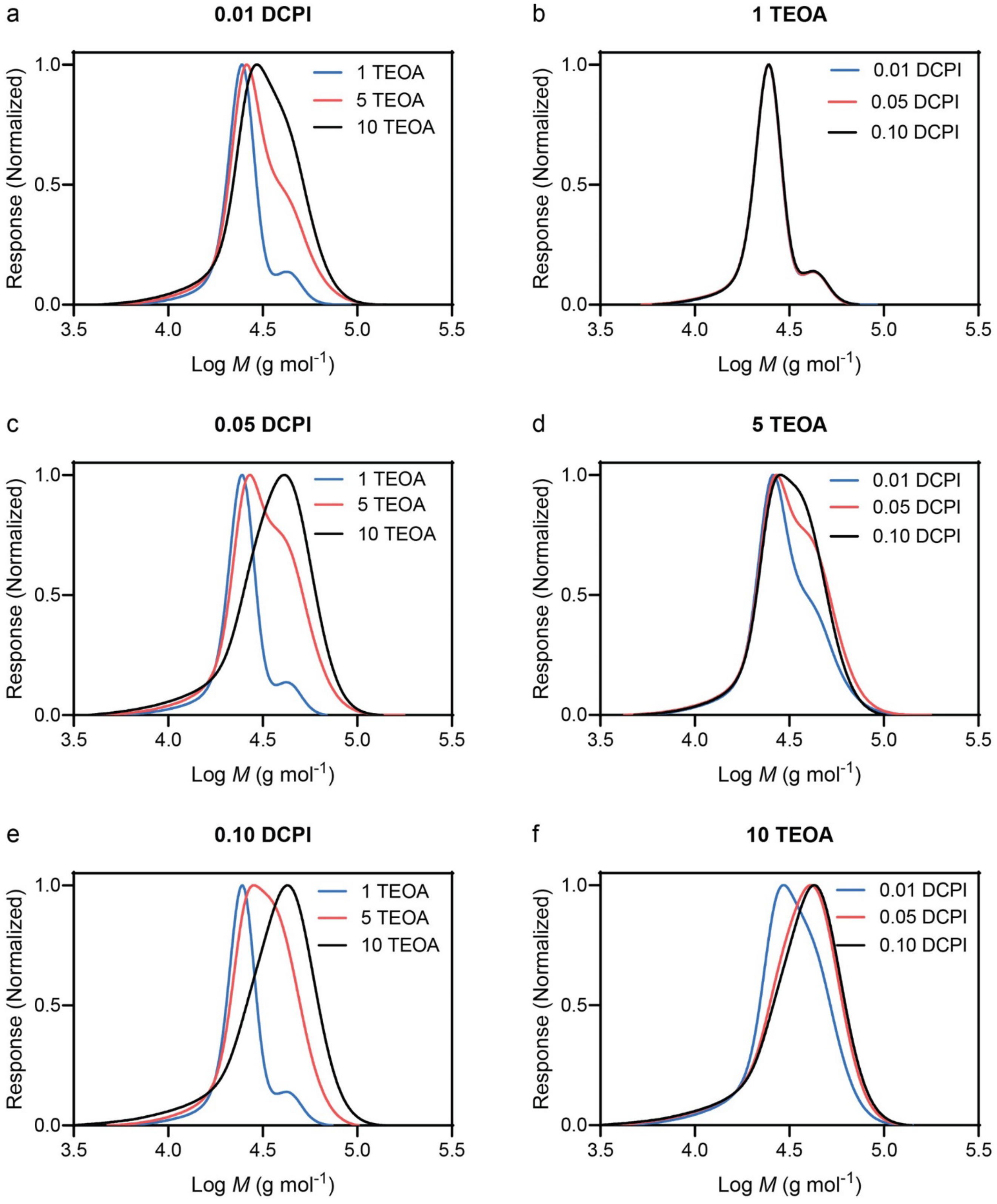

Figure S7. Molecular weight distributions of chain extended linear polymers of PBA_{182}-CTA with DEAm using various weight ratios of DCPI and TEOA on Xube (0.4 mm min^{-1} and 6.4 mW mm^{-2}). Comparison of chain extension efficiency with varying the weight ratio of TEOA from 1 to 5 and to 10 at a fixed weight ratio of DCPI with (a) 0.01, (c) 0.05, (e) 0.10; Comparison of chain extension efficiency with varying the mass ratio of DCPI from 0.01, to 0.05 and to 0.10 at a fixed mass ratio of TEOA with (b) 1, (d) 5, (f) 10.

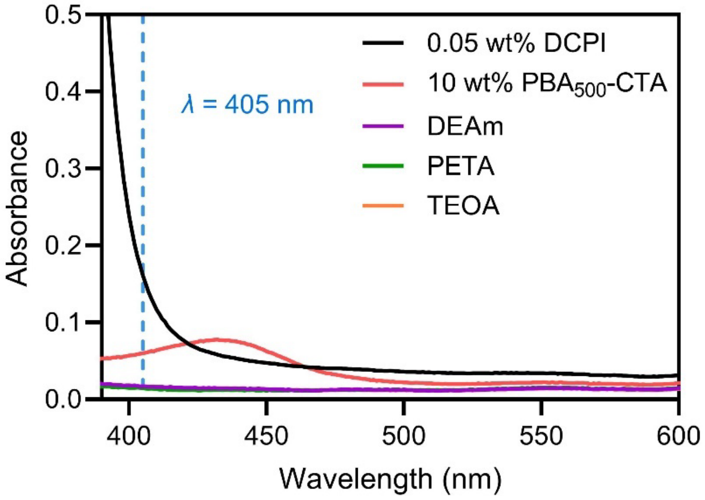

Figure S8. UV-Vis spectra of each component in a typical resin formulation, showing the light absorption of DEAm, PETA, TEOA, 0.05 wt% DCPI in DEAm and 10 wt% PBA_{500}-CTA in DEAm within the visible light range. The optical path length is 10 mm.

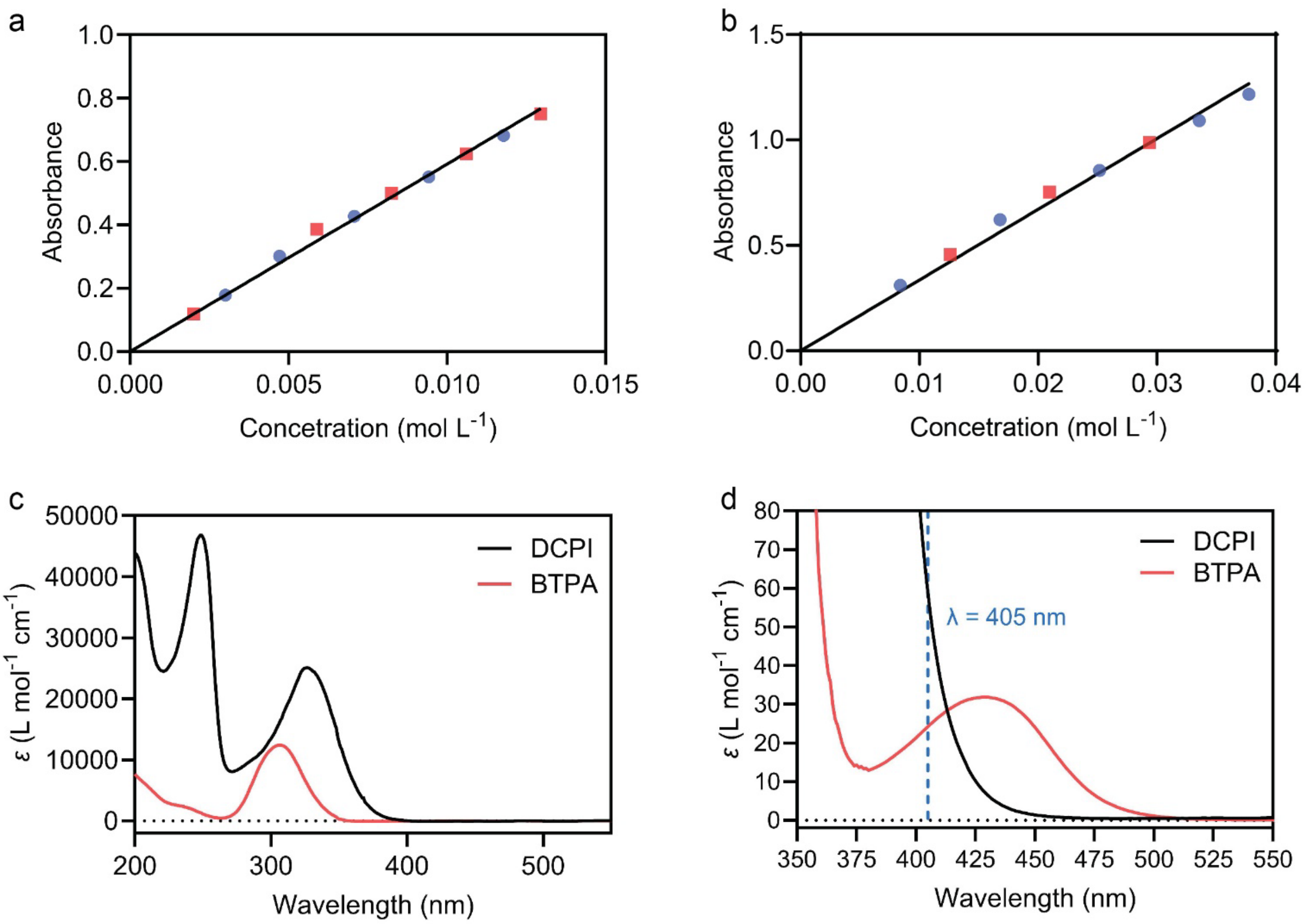

Figure S9. Molar extinction coefficient (ε) of DCPI and BTPA in acetonitrile. Calibration of molar extinction coefficient of: (a) DCPI at 405 nm and (b) BTPA at 430 nm in acetonitrile using two series of constantly diluted samples for each experiment (bule circle and red square). (c) The profile of molar extinction coefficient versus wavelength of DCPI and BTPA in acetonitrile across a broad range, (d) Insight into molar extinction coefficient at 405 nm (blue dash line) corresponding to the wavelength of the laser light-sheet for xolography. ε_{405} (DCPI) = 59 L mol^{-1} cm^{-1}, ε_{405} (BTPA) = 24 L mol^{-1} cm^{-1}, calculated via **Equation S2 (Supporting Information)**.

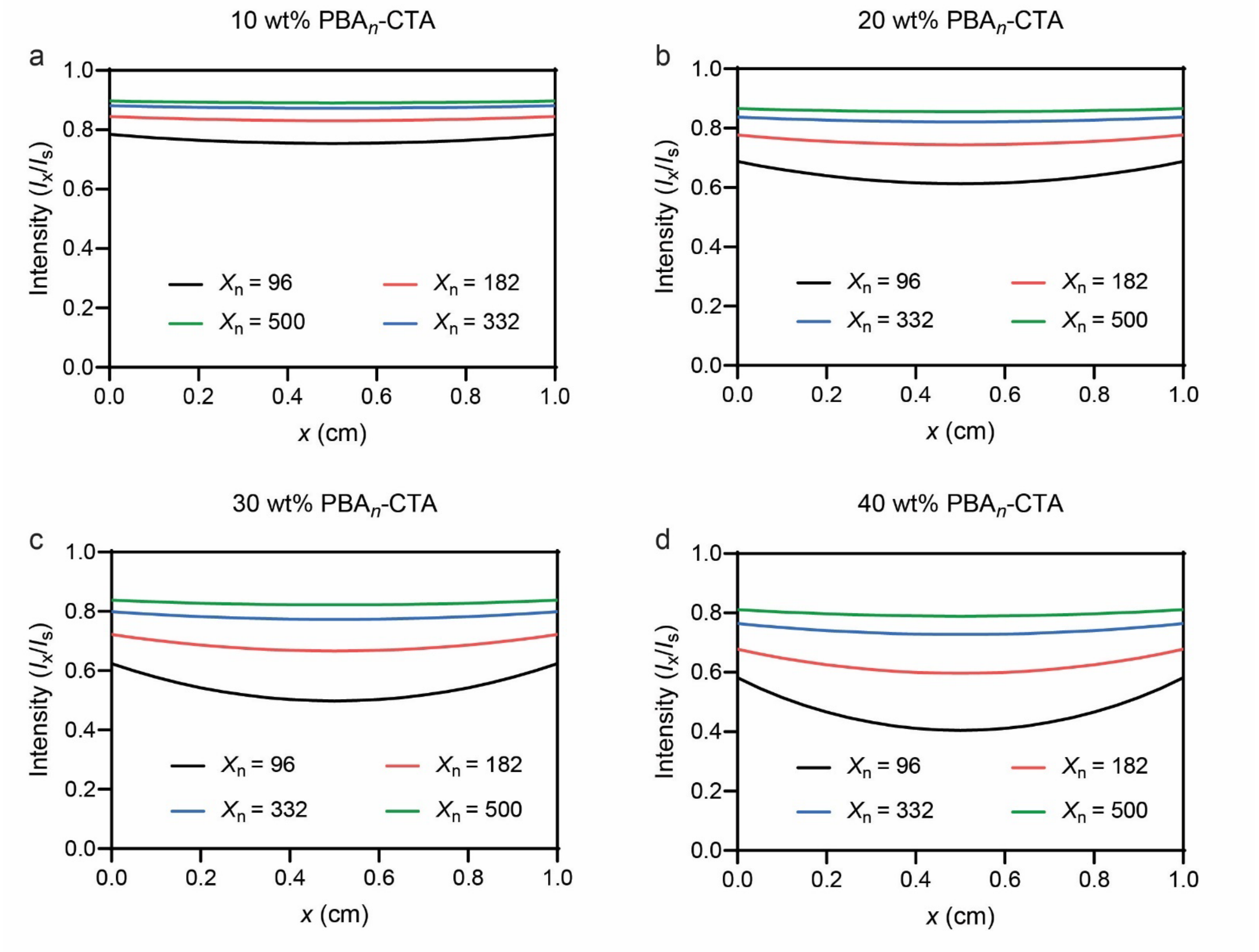

Figure S10. Light distribution profiles of all resin formulations (**Table S5, Supporting Information**). Resins consist of (a) 10 wt% PBA$_n$-CTA, (b) 20 wt% PBA$_n$-CTA, (c) 30 wt% PBA$_n$-CTA and (d) 40 wt% PBA$_n$-CTA, while keeping a mass ratio of DEAm:PETA at 2:1, with 5 wt% TEOA and 0.05 wt% DCPI. All data were calculated based on **Equation S2 (Supporting Information)**, where ε_{405} (DCPI) = 59 L mol^{-1} cm^{-1}, ε_{405} (BTPA) = 24 L mol^{-1} cm^{-1}.

Table S4. Maximum light intensity deviation within the print vat.

Resin[a]	Loading of PBA$_n$-CTA (wt%)	Light intensity attenuation (%)[b]
**M96-10**	10	3.9
**M182-10**		1.7
**M322-10**		0.9
**M500-10**		0.7
**M96-20**	20	10.9
**M182-20**		4.2
**M322-20**		1.9
**M500-20**		1.2
**M96-30**	30	20.2
**M182-30**		7.7
**M322-30**		3.2
**M500-30**		1.9
**M96-40**	40	30.4
**M182-40**		12.0
**M322-40**		4.9
**M500-40**		2.7

[a] -The naming convention is as follows: MXXX-YY, where XXX indicates the degree of polymerization of the PBA$_n$-CTA and YY indicates the loading (wt%) of PBA$_n$-CTA in the resin, with a fixed mass ratio of DEAm:PETA of 2:1, 5wt% TEOA and 0.05 wt% DCPI. [b]-Light intensity attenuation (I_a) was calculated based on **Equation S12 (Supporting Information)**:

$$I_a = \frac{I_0 - I_{0.5}}{I_0} \cdot 100\% \quad \textbf{(S12)}$$

where I_0 and $I_{0.5}$ indicate light intensity (405 nm) where the optical path length is 0 cm and 0.5 cm in the print vat (cuvette), respectively.

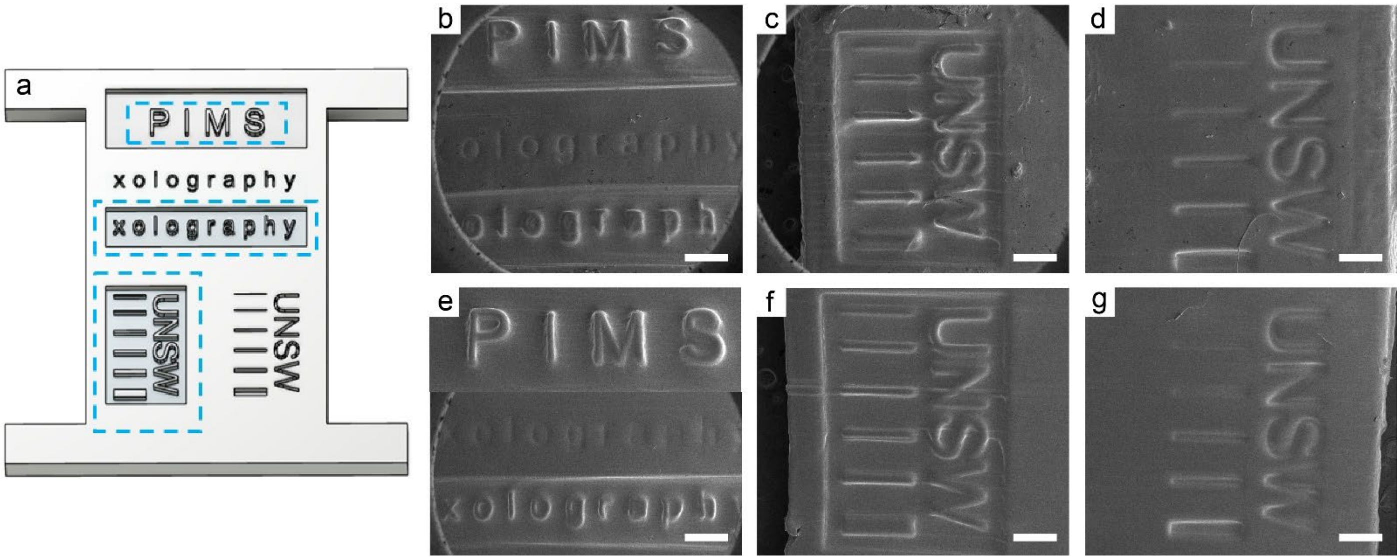

Figure S11. SEM micrographs of materials 3D-printed with various mass ratios of DEAm to PETA using 30 wt% PBA_{332}-CTA, 5 wt% TEOA and 0.05 wt% DCPI, as well as an overall 65 wt% of monomer and crosslinker. (a) The designed model for resolution test, with blue areas indicating positive (protruding) features and others denoting negative (sunk) features and the designed line widths for both positive and negative features are 20, 40, 60, 80, 100 and 200 µm in a top-down sequence. SEM images of objects printed using various mass ratios of DEAm to PETA at: (b-d) 1.6:1 and (e-g) 3.3:1. Scale bars are 500 µm.

Notes: After the measurement of calibrated lines with ImageJ software, the object printed from 1.6:1 mass ratio of DEAm to PETA resin retained a good feature fidelity of 80 µm positive features and 80 µm negative features. For the other one fabricated from the resin consisting of 3.3:1 mass ratio of DEAm to PETA, it showed a better resolution of 60 µm positive features and 80 um negative features.

Table S5. Resins formulated by varying loading and chain length of PBA_n-CTA with a fixed mass ratio of DEAm:PETA of 2:1.

Resin[a]	X_n of PBA_n-CTA	weight percentage of components (wt%)				
		**PBA_n-CTA**	**DEAm**	**PETA**	**TOEA**	**DCPI**
**M96-10**	96	10.00	56.67	28.33	5.00	0.05
**M182-10**	182					
**M322-10**	322					
**M500-10**	500					
**M96-20**	96	20.00	50.00	25.00	5.00	0.05
**M182-20**	182					
**M322-20**	322					
**M500-20**	500					
**M96-30**	96	30.00	43.33	21.67	5.00	0.05
**M182-30**	182					
**M322-30**	322					
**M500-30**	500					
**M96-40**	96	40.00	36.67	18.33	5.00	0.05
**M182-40**	182					
**M322-40**	322					
**M500-40**	500					

[a]-The naming convention is as follows: MXXX-YY, where XXX indicates the degree of polymerization of the PBA_n-CTA and YY indicates the loading (wt%) of PBA_n-CTA in each resin, with a fixed mass ratio of DEAm:PETA of 2:1, 5wt% TEOA and 0.05 wt% DCPI

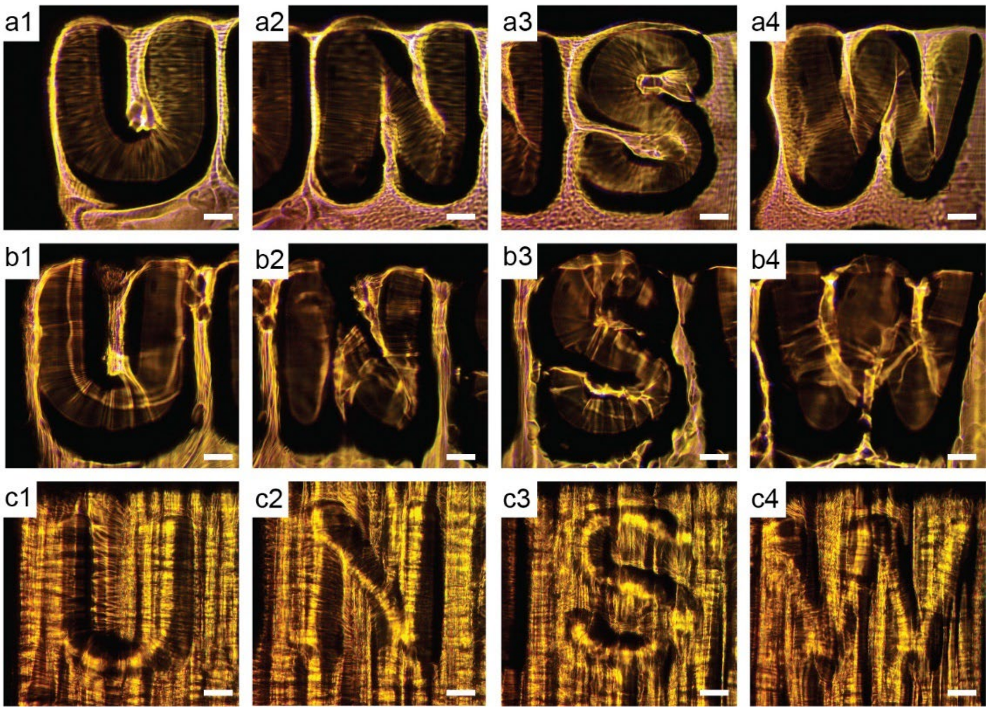

Figure S12. Optical microscope images of samples printed with M500-30 resin under various energy doses (**Equation S13, Supporting Information**). Three objects were fabricated under (a) 0.8 mm min^{-1} and 2.4 mW mm^{-2} (0.9 mJ mm^{-3}); (b) 0.8 mm min^{-1} and 4.8 mW mm^{-2} (1.8 mJ mm^{-3}); (c) 0.8 mm min^{-1} and 9.6 mW mm^{-2} (3.6 mJ mm^{-3}). All samples were post-cured by a 405 nm LED lamp for 5 min. Scale bars: 100 µm.

Equation S13 (Supporting Information) shows the energy does during xolography as a function of light-sheet intensity, speed, and optical path length:

$$\text{Energy dose} = 3 \cdot \frac{\text{Intensity}}{\text{Speed}\cdot l} \quad \textbf{(S13)}$$

where intensity (mW mm^{-2}) represents the power of 405 nm laser light-sheet radiating the resin vat per square millimeter; speed (mm min^{-1}) is the moving rate of the print vat; l (10 mm) is the optical path length of the laser light sheet passing through the resin; energy dose (mJ mm^{-3}) is the energy dose per unit volume of resin receives during the printing process; the thickness of light sheet is assumed to be 50 µm (See more details in **Figure S2c, Supporting Information**).

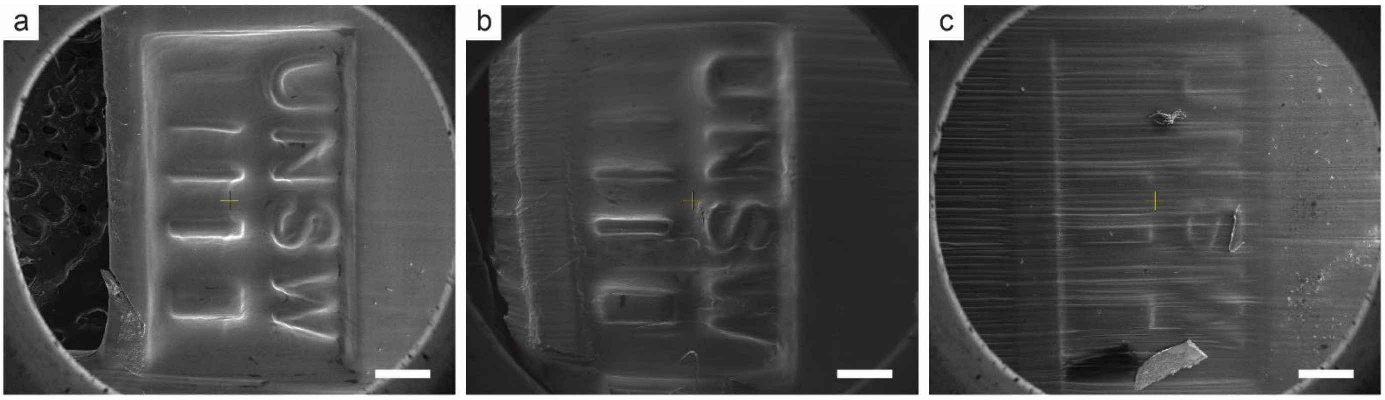

Figure S13. SEM images of objects printed using the M500-30 formulation. Three samples were printed under: (a) 0.8 mm min^{-1} and 2.4 mW mm^{-2}; (b) 0.8 mm min^{-1} and 4.8 mW mm^{-2}; (c) 0.8 mm min^{-1} and 9.6 mW mm^{-2}; The lateral widths of lines from top to bottom are: 20, 40, 80, 160, and 320 µm, respectively. All samples were post-cured under 405 nm irradiation for 5 min (monomer conversion > 90%). Scale bars are 500 µm.

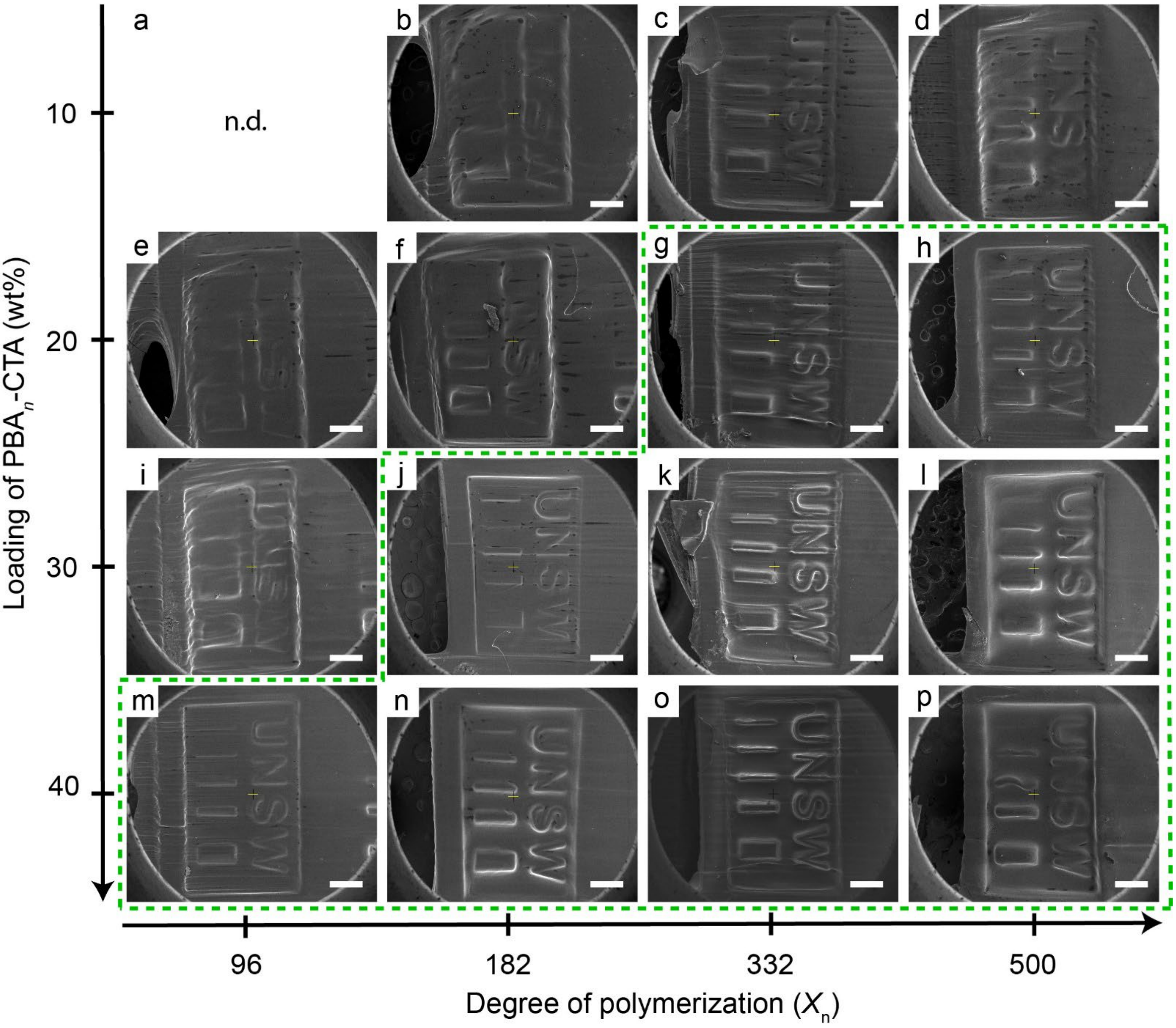

Figure S14. SEM micrographs of positive features of PIMS materials printed under the optimal parameters. Objects were fabricated using (a-d) 10 wt% of PBA$_n$-CTA with X_n = (a) 96 (partially cured), (b) 182, (c) 332, (d) 500; (e-h) 20 wt% of PBA$_n$-CTA with X_n = (e) 96, (f) 182, (g) 332, (h) 500; (i-l) 30 wt% of PBA$_n$-CTA with X_n = (i) 96, (j) 182, (k) 332, (l) 500; (m-p) 40 wt% of PBA$_n$-CTA with X_n = (m) 96, (n) 182, (o) 332, (p) 500. The lateral widths of lines from top to bottom are: 20, 40, 80, 160, and 320 μm, respectively. Scale bars are 500 μm. All samples within the green area indicate fine details, defined in **Table S6** (**Supporting Information**).

Table S6. Measurements of positive details of printed objects from SEM images.

Sample ID	lateral width (µm)					resolution[b]
	line 1	line 2	line 3	line 4	line 5	
**Model[a]**	20	40	80	160	320	-
**M96-10**	-	-	-	-	-	poor
**M96-20**	-	-	-	-	-	poor
**M96-30**	-	-	-	-	-	poor
**M96-40**	60	81	81	156	314	good
**M182-10**	-	-	-	-	-	poor
**M182-20**	-	-	-	-	-	poor
**M182-30**	52	79	86	162	295	good
**M182-40**	68	78	87	173	319	good
**M332-10**	-	-	-	-	-	poor
**M332-20**	55	75	83	162	301	good
**M332-30**	60	68	84	172	300	good
**M332-40**	56	60	77	148	316	good
**M500-10**	-	-	-	-	-	poor
**M500-20**	-	73	74	158	295	good
**M500-30**	-	62	83	154	317	good
**M500-40**	-	66	74	165	318	good

[a]-The positive line bars in the digital model were designed with various lateral widths, increasing from 20 µm for line 1 to 320 µm for line 5. [b]-Resolution is determined by positive features as visualized in SEM micrographs (**Figure S14, Supporting Information**): samples that showed line widths within ± 10% of their target values for 80, 160, and 320 µm lines were defined as having good resolution.

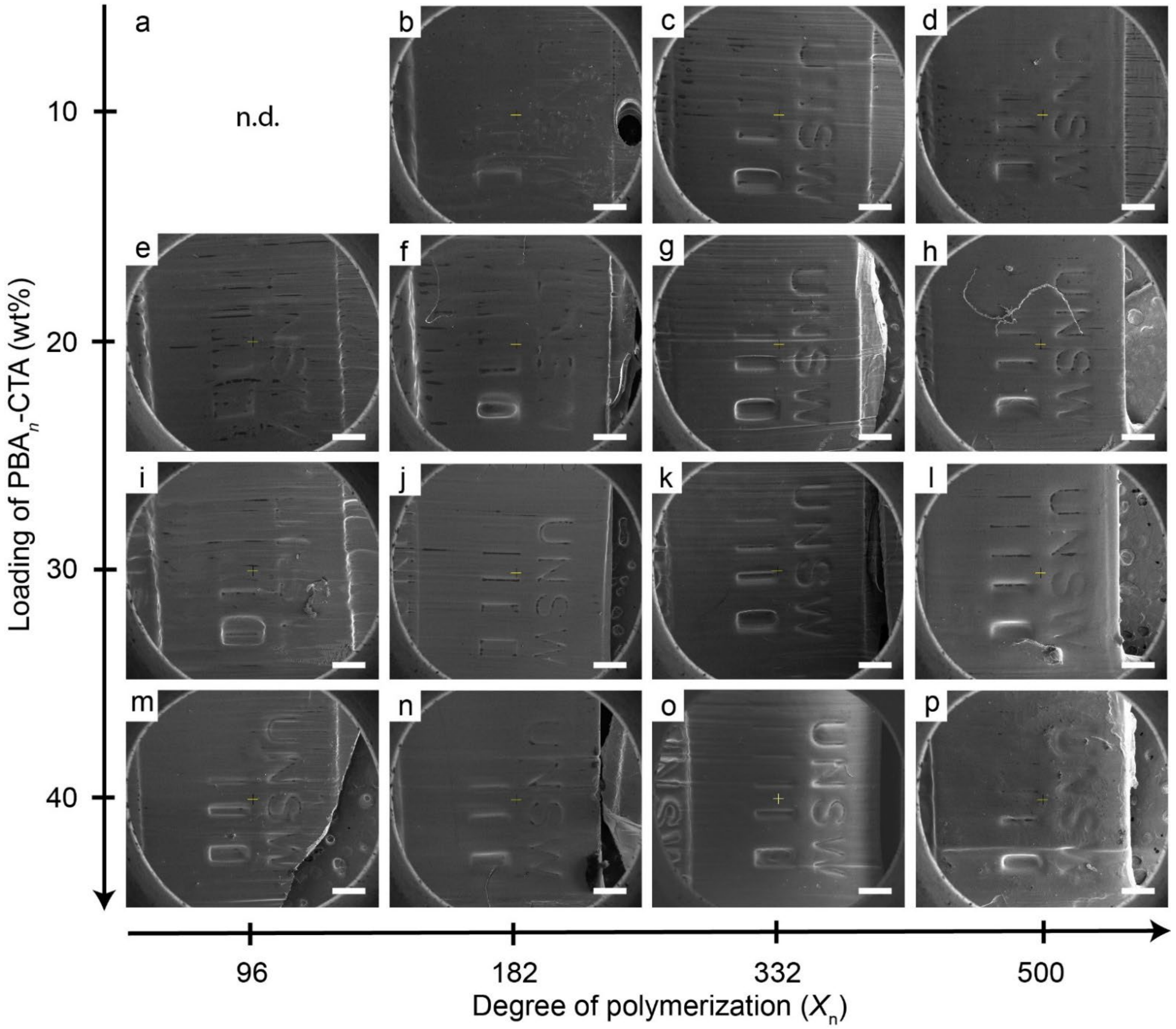

Figure S15. SEM micrographs of negative features of PIMS materials printed under the optimal parameters. Objects were fabricated using (a-d) 10 wt% of PBA$_n$-CTA with X_n = (a) 96 (partially cured), (b) 182, (c) 332, (d) 500; (e-h) 20 wt% of PBA$_n$-CTA with X_n = (e) 96, (f) 182, (g) 332, (h) 500; (i-l) 30 wt% of PBA$_n$-CTA with X_n = (i) 96, (j) 182, (k) 332, (l) 500; (m-p) 40 wt% of PBA$_n$-CTA with X_n = (m) 96, (n) 182, (o) 332, (p) 500. The lateral widths of lines from top to bottom are: 20, 40, 80, 160 and 320 μm, respectively. Scale bars are 500 μm.

Table S7. Measurements of negative details of printed objects from SEM images.

Sample ID	lateral width (µm)					resolution[b]
	line 1	line 2	line 3	line 4	line 5	
**model[a]**	20	40	80	160	320	-
**M96-10**	-	-	-	-	-	poor
**M96-20**	-	-	-	-	-	poor
**M96-30**	-	-	-	-	-	poor
**M96-40**	-	-	73	163	313	good
**M182-10**	-	-	-	-	-	poor
**M182-20**	-	-	-	-	-	poor
**M182-30**	-	36	81	155	292	good
**M182-40**	-	-	80	108	242	poor
**M332-10**	-	-	-	-	-	poor
**M332-20**	-	-	64	200	324	poor
**M332-30**	-	61	85	169	310	good
**M332-40**	27	43	73	169	328	good
**M500-10**	-	-	-	-	-	poor
**M500-20**	-	-	72	160	332	good
**M500-30**	-	38	79	150	306	good
**M500-40**	-	-	-	-	246	poor

[a]-The negative line bars were designed with various lateral widths, increasing from 20 µm for line 1 to 320 µm for line 5. [b]-Resolution is determined by negative features as visualized in SEM micrographs (**Figure S15, Supporting Information**): samples that showed line widths within ± 10% of their target values for 80, 160, and 320 µm lines were defined as having good resolution.

Table S8. Resolution-related factors of 3D-printed samples.

Resin	Loading of PBA$_n$-CTA (wt%)	X_n of PBA$_n$-CTA[a]	Viscosity (mPa s)[b]	Half-life (s)[c]	Conversion (%)[d]	Resolution[e]
**M96-10**	10	96	12.56	5.8	91	Poor
**M182-10**		182	16.00	4.5	92	Poor
**M322-10**		322	23.25	4.3	93	Poor
**M500-10**		500	28.37	4.4	92	Poor
**M96-20**	20	96	29.21	4.8	90	Poor
**M182-20**		182	44.65	4.3	93	Poor
**M322-20**		322	73.48	4.2	94	Good
**M500-20**		500	115.50	4.3	92	Good
**M96-30**	30	96	64.74	4.6	93	Poor
**M182-30**		182	116.80	4.3	92	Good
**M322-30**		322	177.70	4.2	94	Good
**M500-30**		500	323.70	4.3	91	Good
**M96-40**	40	96	134.50	4.0	95	Good
**M182-40**		182	285.60	4.4	91	Good
**M322-40**		322	570.20	4.0	96	Good
**M500-40**		500	1034.00	4.4	91	Good

[a]-X_n of PBA$_n$-CTA was determined via ^{1}H NMR. [b,c]-All resins were prepared based on the formulation (**Table S5, Supporting Information**) without TEOA to avoid gelation during the measurement. [d]-The conversion of vinyl bonds of printed objects after post-curing under violet light (λ_{max} = 405 nm, 166 mW cm^{-2}) for 5 min, measured by FTNIR. [e]-Samples that showed line widths within ± 10% of their target values for 80, 160, and 320 μm lines were defined as having good resolution (see details in **Table S6, Supporting Information**).

Table S9. Molar ratio of [DEAm]:[PETA]:[BA] per macromolecular chain for various resin formulations.

Resin	loading (wt%)	X_n	Molar ratio			Number of propagating chains (L^{-1})[a]	conversion (%)	N_{total}[b]	χN[c]
			DEAm	PETA	PBA$_n$-CTA				
**M96-10**	10	96	559	101	1	4.80×10^{21}	91	697	127
**M182-10**		182	1050	189	1	2.55×10^{21}	92	1322	241
**M322-10**		322	1907	344	1	1.41×10^{21}	93	2415	441
**M500-10**		500	2866	517	1	9.36×10^{20}	92	3612	659
**M96-20**	20	96	247	44	1	9.60×10^{21}	90	358	65
**M182-20**		182	463	84	1	5.11×10^{21}	93	691	126
**M322-20**		322	841	152	1	2.81×10^{21}	94	1255	229
**M500-20**		500	1264	228	1	1.87×10^{21}	92	1873	342
**M96-30**	30	96	142	26	1	1.44×10^{22}	93	252	46
**M182-30**		182	268	48	1	7.66×10^{21}	92	473	86
**M322-30**		322	486	88	1	4.22×10^{21}	94	862	157
**M500-30**		500	730	132	1	2.81×10^{21}	91	1284	234
**M96-40**	40	96	90	16	1	1.92×10^{22}	95	197	36
**M182-40**		182	170	31	1	1.02×10^{22}	91	365	67
**M322-40**		322	308	56	1	5.63×10^{21}	96	671	123
**M500-40**		500	464	84	1	3.74×10^{21}	91	999	182

[a]-The number of propagating chains per volume was evaluated using **Equation S14 (Supporting Information)**:

$$Number\ of\ chains\ per\ liter = \frac{m}{M_n \times V} \times N_A \qquad \textbf{(S14)}$$

where m (g) is mass of PBA$_n$-CTA in the resin; M_n is the number-average molecular weight of PBA$_n$-CTA determined by ^{1}H NMR; V is total volume of resin (L); N_A is Avogadro number (6.02×10^{23} mol^{-1}). [b]-$N_{total} = N_{PBA\text{-}CTA} + N_{net\text{-}P(DEAm\text{-}stat\text{-}PETA)}$. [c]-Evaluated $\chi = 0.1825$ (**Equation S9, Supporting Information**).

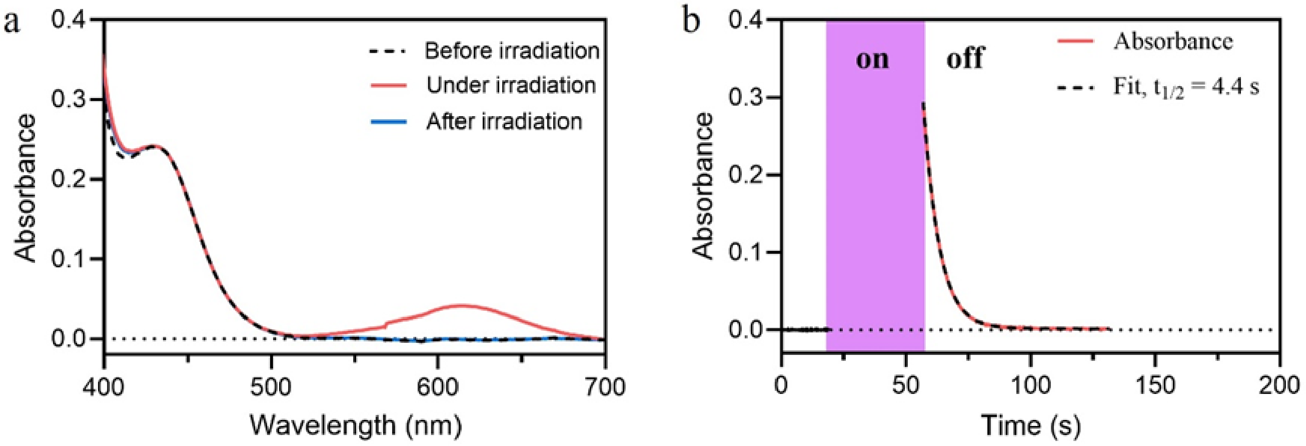

Figure S16. Characteristic UV spectra of photo-sensitive DCPI. (a) Absorbance spectra of DCPI in a typical resin with and without 405 nm irradiation. (b) Decay profile of metastable merocyanine state in the dark after the irradiation of violet light (405 nm). Resin was prepared with 42.11 wt% PBA_{500}-CTA, 38.60 wt% DEAm, 19.30 wt% PETA and 0.05 wt% DCPI without the addition of TEOA, which is similar to the M500-40 but without TEOA.

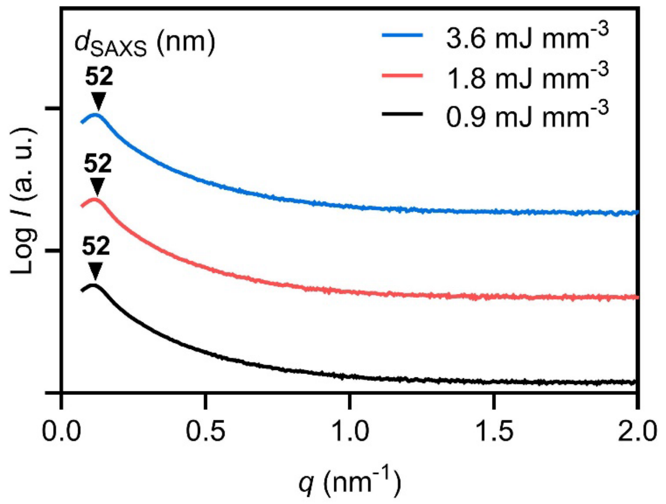

Figure S17. The SAXS profile of objects printed under various conditions using M500-30 formulation. Three samples were fabricated under: (a) 0.8 mm min^{-1} and 2.4 mW mm^{-2} (0.9 mJ mm^{-3}); (b) 0.8 mm min^{-1} and 4.8 mW mm^{-2} (1.8 mJ mm^{-3}); (c) 0.8 mm min^{-1} and 9.6 mW mm^{-2} (3.6 mJ mm^{-3}). All samples were post-cured under 405 nm irradiation for 5 min.

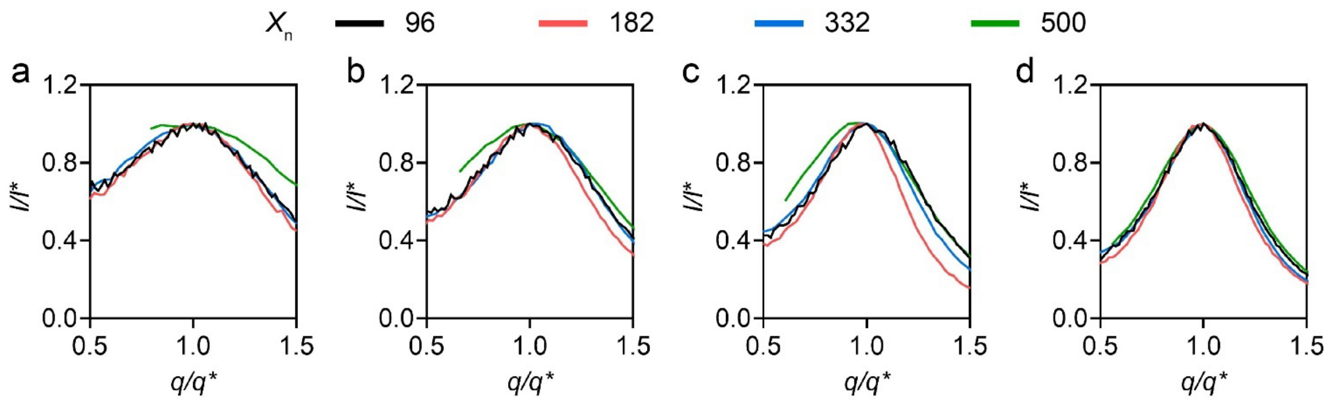

Figure S18. Comparison of the breadths of SAXS peaks for samples 3D-printed using (a) 10 wt%, (b) 20 wt%, (c) 30 wt% and (d) 40 wt% PBA$_n$-CTA, with X_n varying from 96 to 500.The SAXS spectra were normalized based on the intensity (I^*) and the position (q^*) of the principal peaks.

Table S10. Parameters extracted from fitting SAXS data via Teubner-Strey (T-S) model.

**Loading of PBA$_n$-CTA (wt%)**	**X_n of PBA$_n$-CTA**	**d_{SAXS} (nm)**[a]	**d_{TS} (nm)**[f]	**ξ (nm)**[c]	**ξ/d_{TS}**[d]	**a_2**[e]	**c_1**[e]	**c_2**[e]	**f_a**[b]
10	96	23	19	6.8	0.36	36.05	-370	2138	-0.67
	182	32	27	10.1	0.37	42.57	-923	10406	-0.69
	332	47	40	12.8	0.32	25.43	-997	26844	-0.60
	500	66	59	14.9	0.25	12.26	-666	49288	-0.43
20	96	20	18	7.1	0.40	53.01	-532	2541	-0.73
	182	28	25	11.3	0.46	85.80	-1855	16305	-0.78
	332	40	34	14.7	0.43	70.22	-2757	46695	-0.76
	500	58	54	18.5	0.34	32.32	-2523	117135	-0.65
30	96	19	17	8.6	0.51	128.71	-1382	5470	-0.82
	182	26	25	15.1	0.61	240.82	-6165	51989	-0.87
	332	38	36	18.9	0.52	135.57	-6889	127599	-0.83
	500	52	52	21.1	0.40	54.78	-4809	198212	-0.73
40	96	18	17	9.7	0.57	196.16	-2259	8853	-0.86
	182	24	22	13.3	0.62	259.44	-4991	31290	-0.88
	332	35	35	21.2	0.61	250.31	-12423	201996	-0.87
	500	49	48	25.6	0.53	149.56	-13408	429497	-0.84

[a]-Domain spacing determined from SAXS; [b,c,f]-f_a, ξ, and d_{TS} were determined from T-S fitting using **Equations S6-S8 (Supporting Information)**; [d]-The ratio of ξ/d_{TS} indicates the dispersity of domain size; [e]-Parameters calculated based on SAXS fitting using the T-S model.

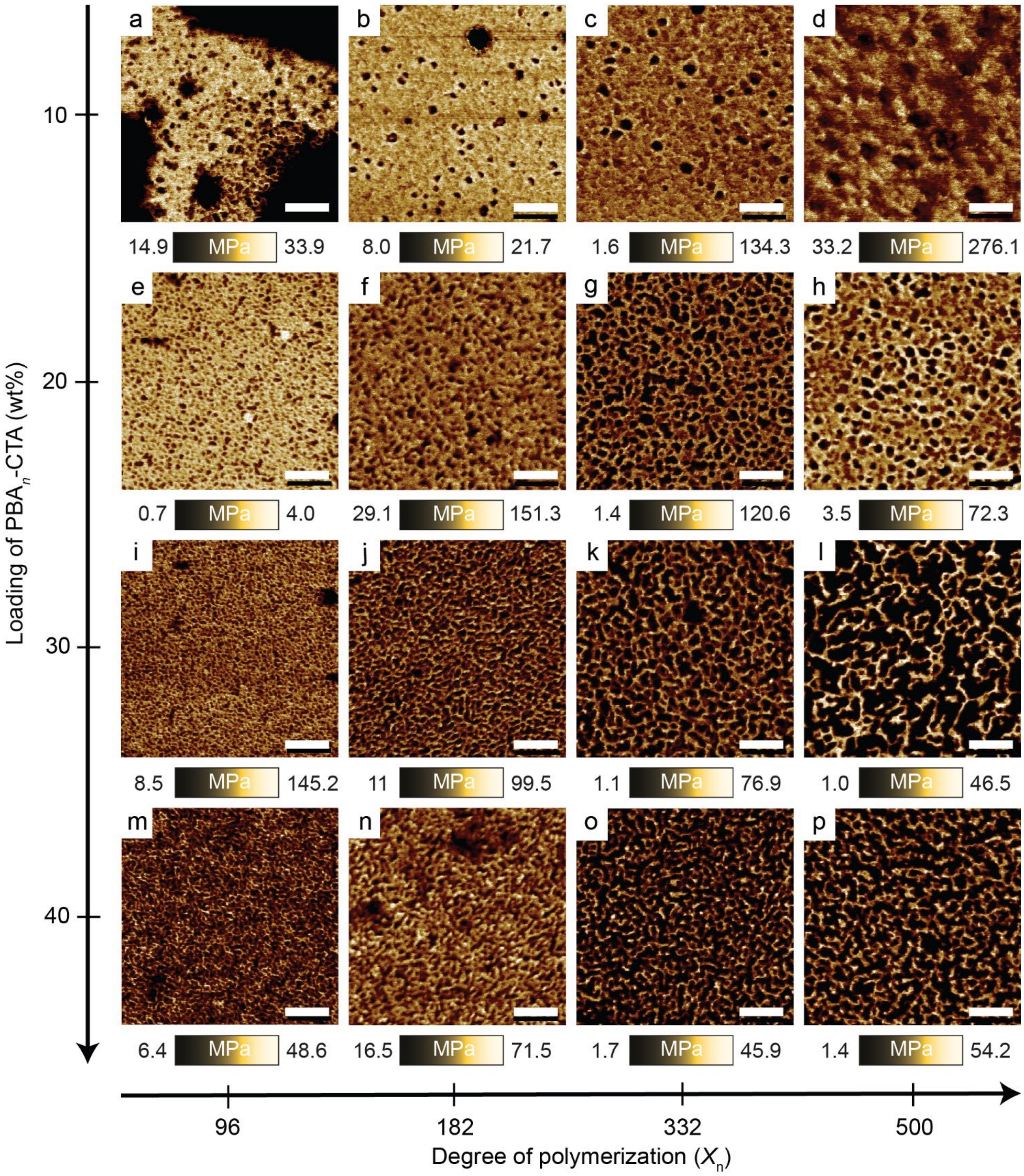

Figure S19. AFM characterization of nanostructured materials prepared via xolography with the lower magnification. These materials were printed using (a-d) 10 wt% PBA$_n$-CTA with X_n = (a) 96, (b) 182, (c) 332, (d) 500; (e-h) 20 wt% PBA$_n$-CTA with X_n = (e) 96, (f) 182, (g) 332, (h) 500; (i-l) 30 wt% PBA$_n$-CTA with X_n = (i) 96, (j) 182, (k) 332, (l) 500 and (m-p) 40 wt% PBA$_n$-CTA with X_n = (m) 96, (n) 182, (o) 332, (p) 500. Samples were printed using a fixed mass ratio of DEAm:PETA of 2:1 with 5 wt% TEOA and 0.05 wt% DCPI. Scale bars are 200 nm.

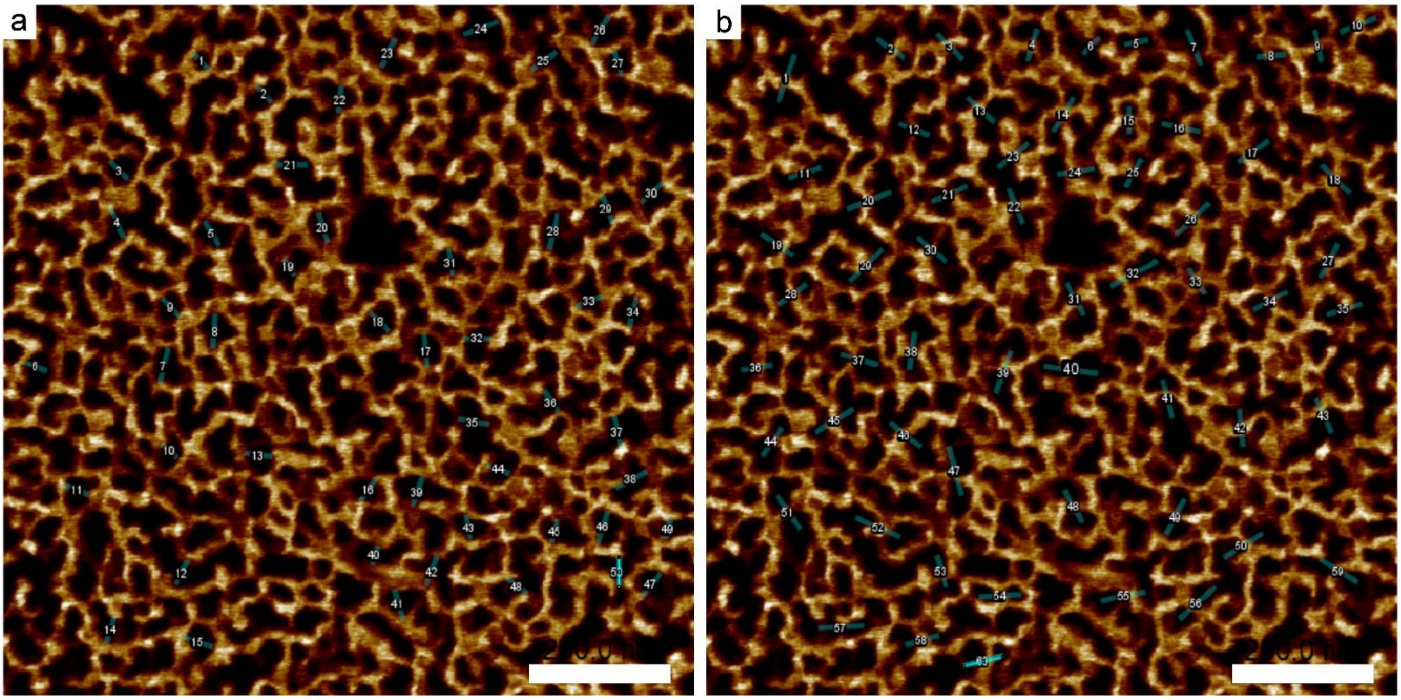

Figure S20. Characteristic illustration of the statistical calculation of nanodomain sizes from the M332-30 fabricated sample: (a) Measurement of PBA$_n$-CTA domain width (D_{PBA}); (b) Measurement of domain spacing (d_{AFM}). Scale bars are 200 nm.

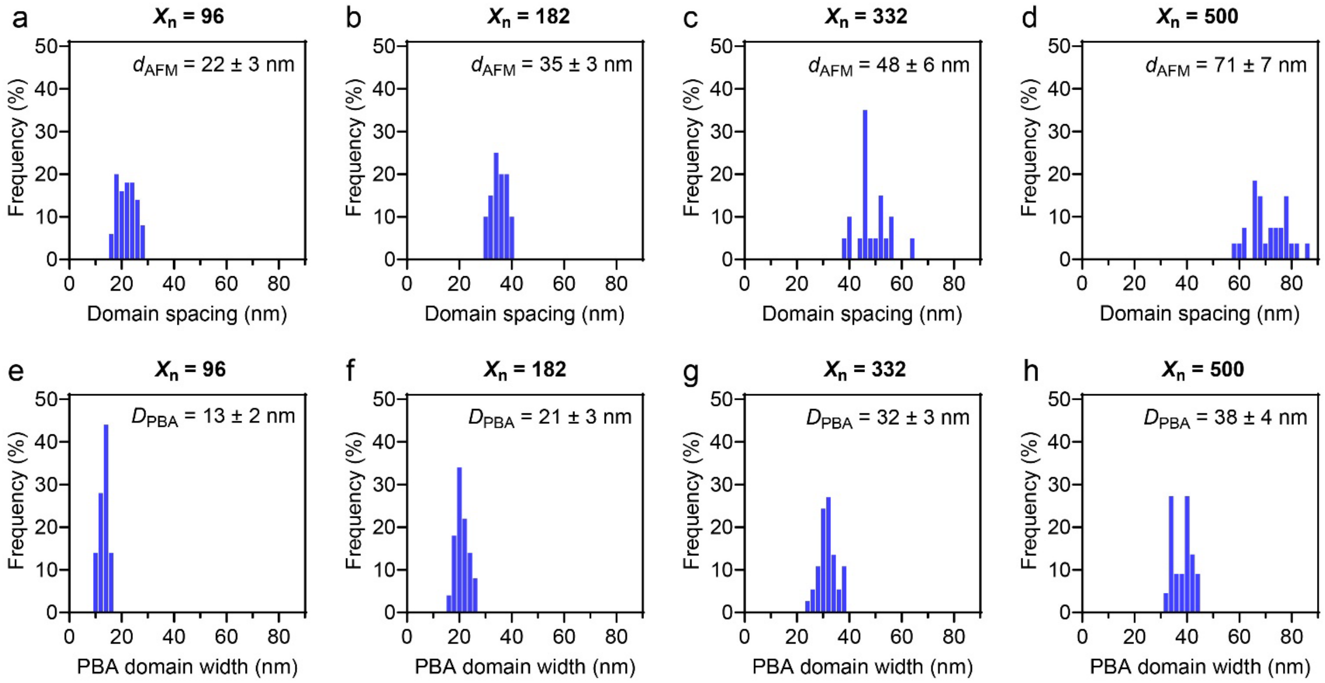

Figure S21. Statistical data of nanodomain sizes of materials printed with various PBA$_n$-CTA chain length (X_n). Distributions of (a-d) domain spacing (d_{AFM}) and (e-h) PBA$_n$-CTA domain width (D_{PBA}), with PBA$_n$-CTA X_n = (a, e) 96, (b, f) 182, (c, g) 332, (d, h) 500. Samples were printed using the formulation with 10 wt% PBA$_n$-CTA, a fixed mass ratio of DEAm:PETA (2:1), 5 wt% TEOA and 0.05 wt% DCPI. The data (d_{AFM} and D_{PBA}) represent the mean ± s.d. of at least 20 measurements for M332-10 and M500-10, and at least 50 measurements for other formulations.

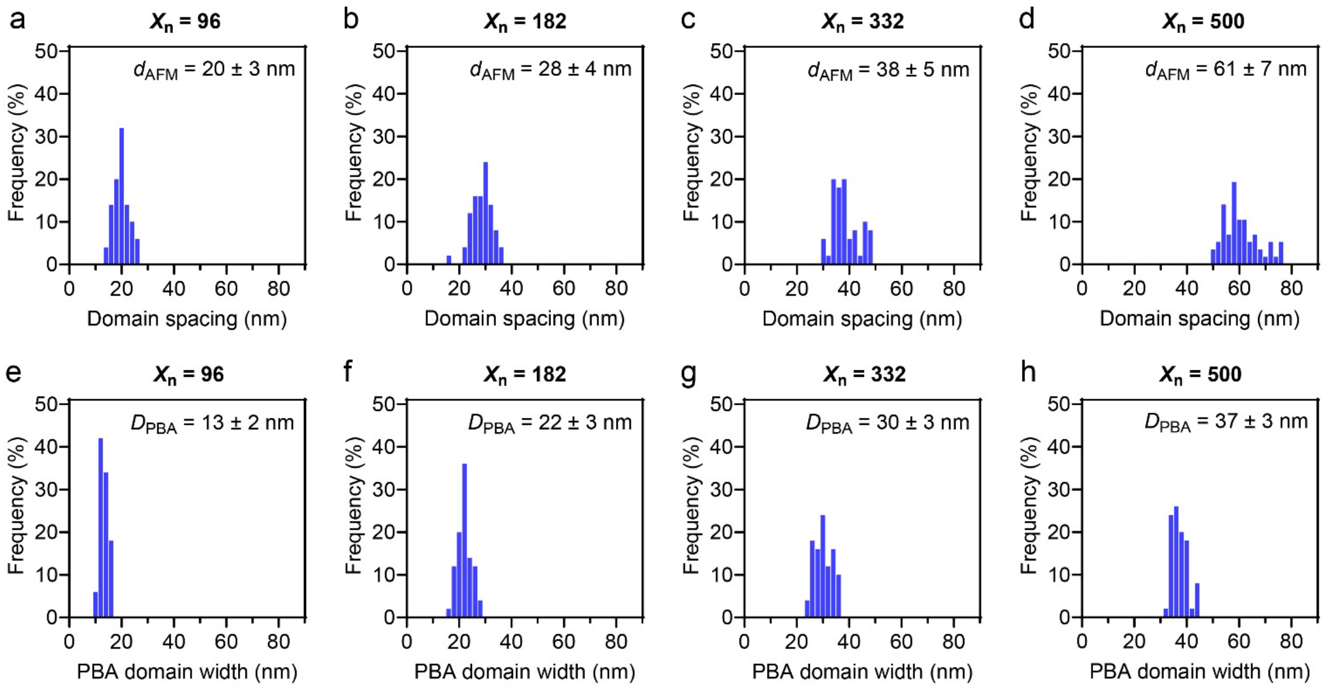

Figure S22. Statistical data of nanodomain sizes of materials printed with various PBA$_n$-CTA chain length (X_n). Distributions of (a-d) domain spacing (d_{AFM}) and (e-h) PBA$_n$-CTA domain width (D_{PBA}), with PBA$_n$-CTA X_n = (a, e) 96, (b, f) 182, (c, g) 332, (d, h) 500. Samples were printed using the formulation with 20 wt% PBA$_n$-CTA, a fixed mass ratio of DEAm:PETA (2:1), 5 wt% TEOA and 0.05 wt% DCPI. The data (d_{AFM} and D_{PBA}) represent the mean ± s.d. of at least 20 measurements for M332-10 and M500-10, and at least 50 measurements for other formulations.

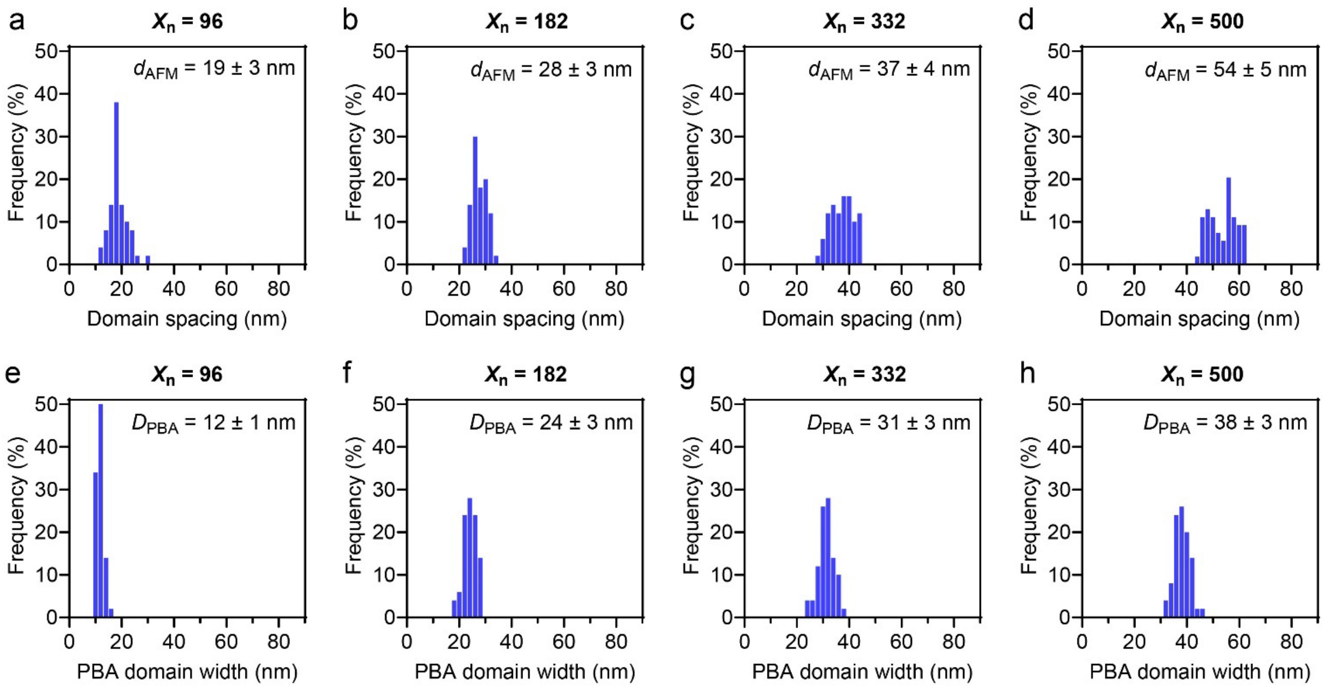

Figure S23. Statistical data of nanodomain sizes of materials printed with various PBA$_n$-CTA chain length (X_n). Distributions of (a-d) domain spacing (d_{AFM}) and (e-h) PBA$_n$-CTA domain width (D_{PBA}), with PBA$_n$-CTA X_n = (a, e) 96, (b, f) 182, (c, g) 332, (d, h) 500. Samples were printed using the formulation with 30 wt% PBA$_n$-CTA, a fixed mass ratio of DEAm:PETA (2:1), 5 wt% TEOA and 0.05 wt% DCPI. The data (d_{AFM} and D_{PBA}) represent the mean ± s.d. of at least 20 measurements for M332-10 and M500-10, and at least 50 measurements for other formulations.

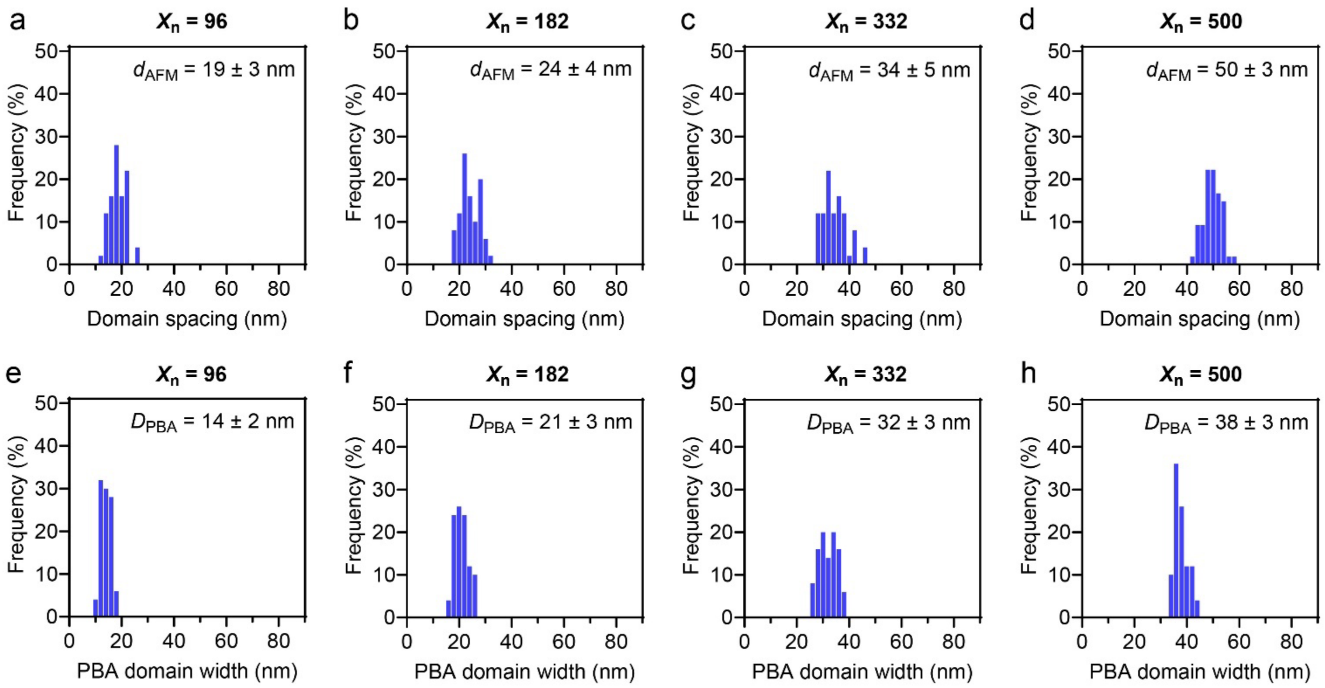

Figure S24. Statistical data of nanodomain sizes of materials printed with various PBA$_n$-CTA chain length (X_n). Distributions of (a-d) domain spacing (d_{AFM}) and (e-h) PBA$_n$-CTA domain width (D_{PBA}), with PBA$_n$-CTA X_n = (a, e) 96, (b, f) 182, (c, g) 332, (d, h) 500. Samples were printed using the formulation with 40 wt% PBA$_n$-CTA, a fixed mass ratio of DEAm:PETA (2:1), 5 wt% TEOA and 0.05 wt% DCPI. The data (d_{AFM} and D_{PBA}) represent the mean ± s.d. of at least 20 measurements for M332-10 and M500-10, and at least 50 measurements for other formulations.

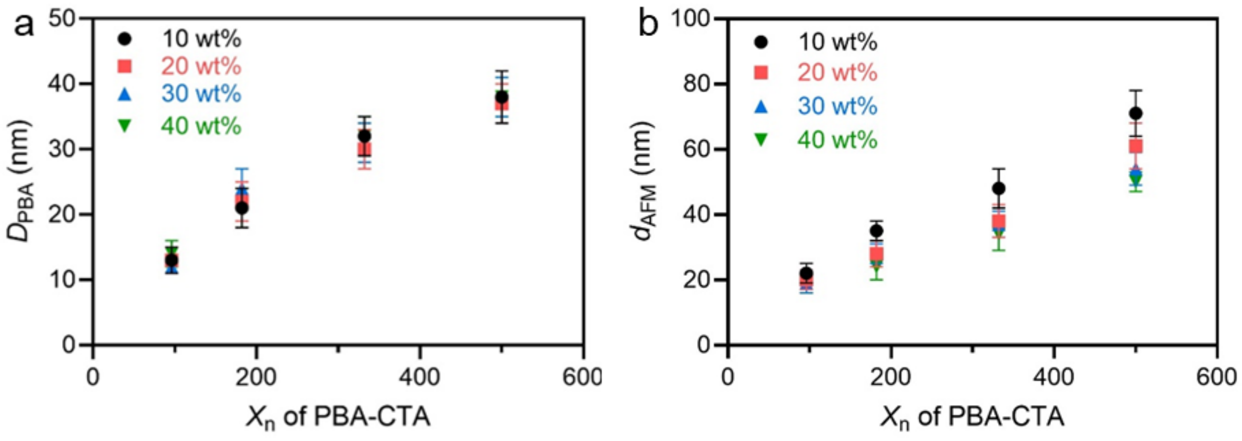

Figure S25. The dependence of domain sizes on the chain length (X_n) and loading (wt%) of PBA$_n$-CTA. (a) The dependence of domain width (D_{PBA}) on PBA$_n$-CTA X_n and mass fraction (wt%). (b) The dependence of domain spacing (d_{AFM}) on PBA$_n$-CTA X_n and mass fraction (wt%). The data (D_{PBA} and d_{AFM}) represent the mean ± s.d. of at least 20 measurements for M332-10 and M500-10, and at least 50 measurements for other formulations.

Table S11. Summary of nanoscale information of materials printed with various formulations.

Resin	loading of PBA_n-CTA (wt%)	X_n of PBA_n-CTA[a]	Morphology			
			Type[b]	D_{PBA} (nm)[c]	d_{AFM} (nm)[c]	d_{SAXS} (nm)[d]
**M96-10**	10	96	Globular	13 ± 2	22 ± 3	23
**M182-10**		182	Globular	21 ± 3	35 ± 3	32
**M322-10**		322	Globular	32 ± 3	48 ± 6	47
**M500-10**		500	Globular	38 ± 4	71 ± 7	66
**M96-20**	20	96	Globular	13 ± 2	20 ± 3	20
**M182-20**		182	Elongated	22 ± 3	28 ± 4	28
**M322-20**		322	Elongated	30 ± 3	38 ± 5	40
**M500-20**		500	Elongated	37 ± 3	61 ± 7	58
**M96-30**	30	96	Elongated	12 ± 1	19 ± 3	19
**M182-30**		182	Bicontinuous	24 ± 3	28 ± 3	26
**M322-30**		322	Bicontinuous	31 ± 3	37 ± 4	38
**M500-30**		500	Bicontinuous	38 ± 3	54 ± 5	52
**M96-40**	40	96	Bicontinuous	14 ± 2	19 ± 3	18
**M182-40**		182	Bicontinuous	21 ± 3	24 ± 4	24
**M322-40**		322	Bicontinuous	32 ± 3	34 ±5	35
**M500-40**		500	Bicontinuous	38 ± 3	50 ± 3	49

[a]-Degree of polymerization (X_n) of PBA_n-CTA determined by ^{1}H NMR. [b]-Morphology of printed objects determined by AFM. [c]-PBA_n-CTA domain witdth (D_{PBA}) and domain spacing (d_{AFM}) calculated by counting at least 50 points from AFM images except M332-10 and M500-10 due to their insufficient points for statistics. [d]-Domain spacing (d_{SAXS}) determined by SAXS. The data (D_{PBA} and d_{AFM}) represent the mean ± s.d. of at least 20 measurements for M332-10 and M500-10, and at least 50 points for other formulations.

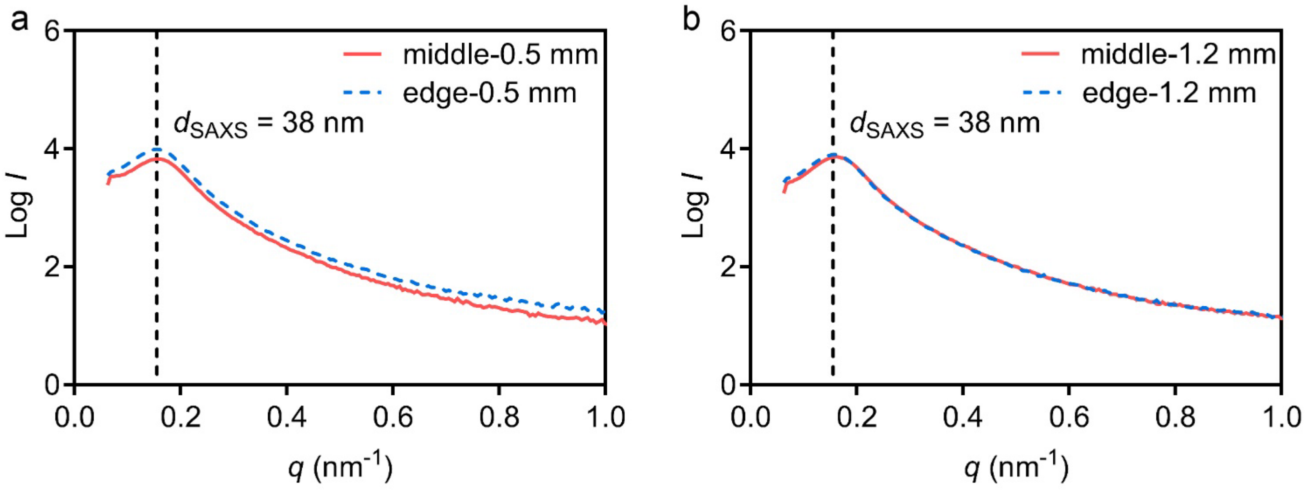

Figure S26. SAXS characterization of nanostructured materials across the macroscale, printed with M332-30 under the same speed and light intensity, followed by a 5-min post-curing process. Spatial nano-structural characterization of two cuboids printed with various thicknesses: (a) 8 × 12 × 0.5 mm and (b) 8 × 12 × 1.2 mm on the middle (red solid line) and edge (blue dash line) regions.

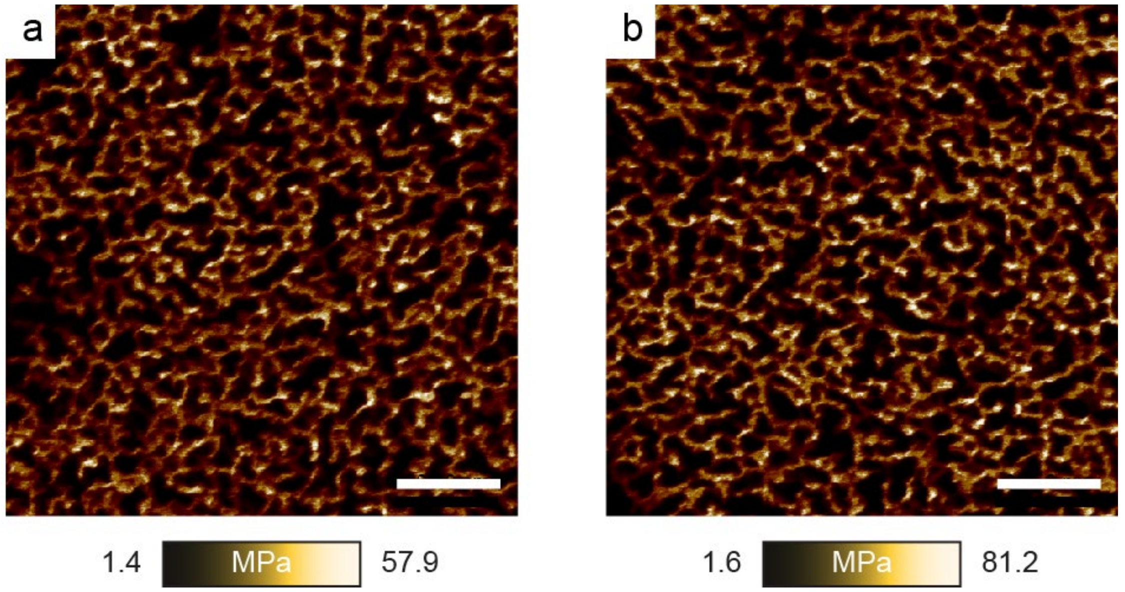

Figure S27. AFM characterization of nanostructured materials (8 × 12 × 2 mm) across the macroscale, printed with M332-30 under the same speed and light intensity, followed by a 5-min post-curing process. (a) The middle and (b) the edge areas were analyzed on the surface of one sample. Domain spacing (d_{AFM}) was measured via ImageJ for counting: (a) d_{AFM} = 38 ± 5 nm; (b) d_{AFM} = 37 ± 6 nm. The data (d_{AFM}) represents the mean ± s.d. of at least 50 measurements.

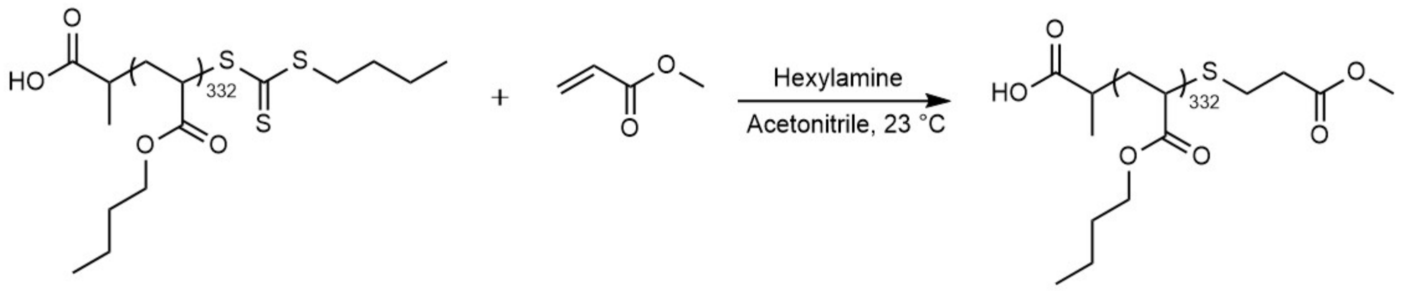

Figure S28. Synthesis of inert PBA_{332} via aminolysis of PBA_{332}-CTA in the presence of methyl acrylate. The method is available in **Aminolysis of PBA_{332}-CTA** section.

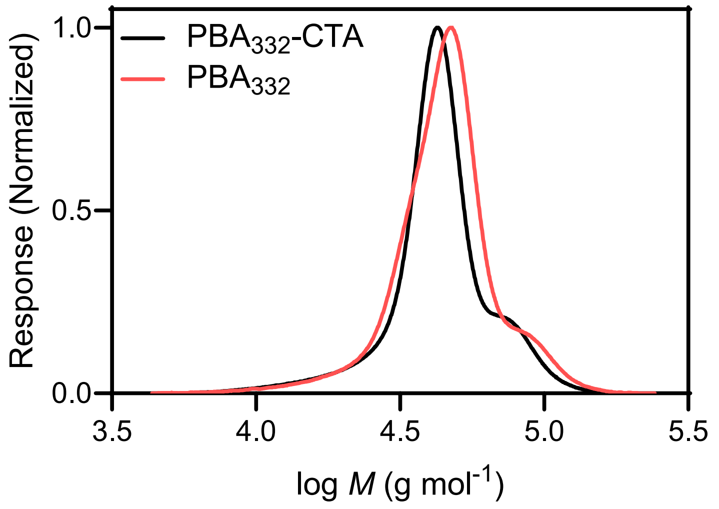

Figure S29. Molecular weight distributions of PBA_{332}-CTA and PBA_{332}. DMAc was used as eluent and PMMA as the calibration standard.

Table S12. Monomer conversion of PIMS and PIPS materials before and after post-curing.

Post-curing time (min)	Monomer conversion (%)[a]	
	M332-40	P332-40[d]
0	42	14
5[b]	91	58
30[b]	-	82
660[b]	-	86
1200[c]	-	90

[a]-Both samples were printed with the same parameters (0.8 mm min^{-1} and 3.2 mW mm^{-2}), followed by the calculation of conversion of vinyl bonds via FTNIR. [b]-405 nm LED lamp (166 mW cm^{-2}) was used for post-curing; [c]-365 nm LED lamp was adopted to further increase monomer conversion of PIPS material to match that of PIMS material for subsequent hardness testing; [d]-PIPS material was irradiated for both sides with the same time (half the total time listed in **Table S12, Supporting Information** on each side) within a total post-curing period considering its opacity. For P332-40, it consists of 40 wt% PBA_{332}, 5 wt% TEOA and 0.05 wt% DCPI while keeping the mass ratio of DEAm to PETA at 2:1.

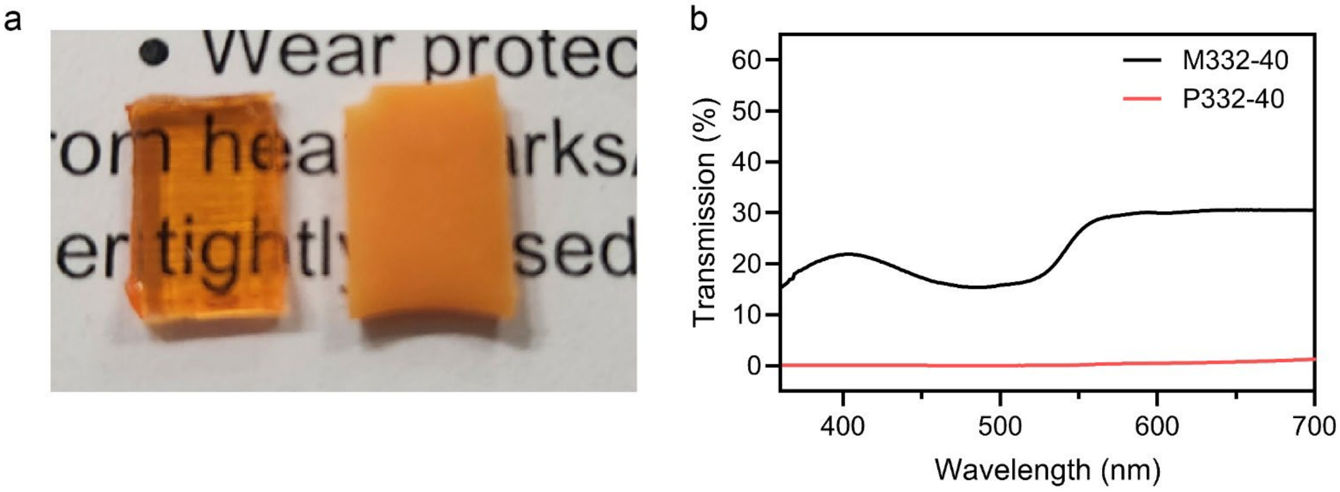

Figure S30. Optical transparency of PIMS and PIPS materials after post-curing. (a) Photographs (from left to right: M332-40, P332-40). (b) UV-Vis spectra of PIMS (M332-40) and PIPS (P332-40) objects (2 mm thickness).

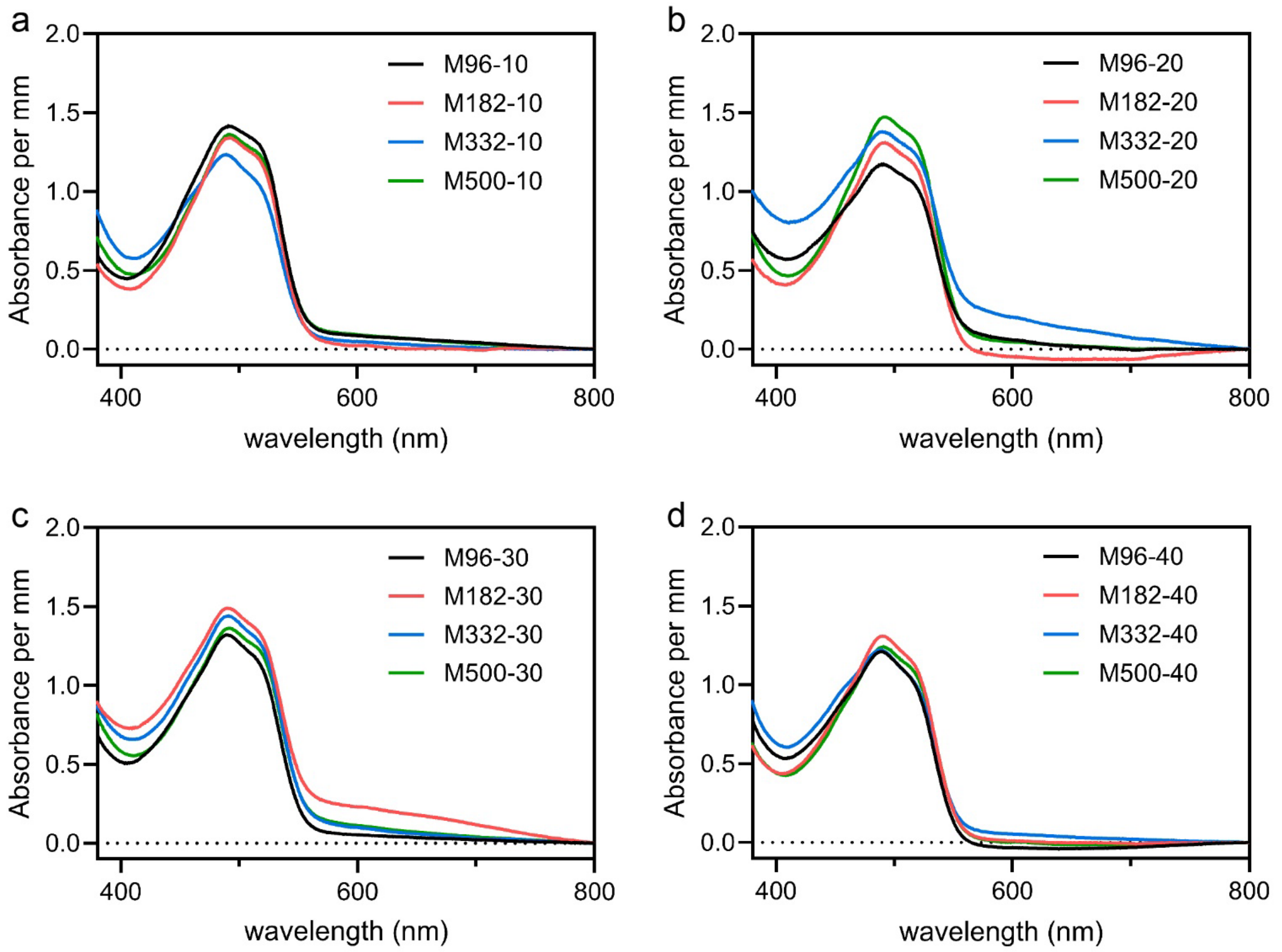

Figure S31. Optical spectra of all PIMS materials (absorbance per millimeter thickness) investigated in this study. Objects were printed using (a) 10 wt%, (b) 20 wt%, (c) 30 wt% and (d) 40 wt% PBA$_n$-CTA of various chain lengths (X_n = 96, 182, 332 and 500). In all cases, the concentrations of TEOA and DCPI were 5 wt% and 0.05 wt%, with a fixed mass ratio of DEAm to PETA of 2:1. The absorbance of materials was zeroed at 800 nm.

Table S13. Hardness values of PIMS and PIPS materials measured by Vickers hardness tester

Sample	M332-40	P332-40
**HV (kgf mm^{-2})^{a}**	0.89 ± 0.03	0.69 ± 0.04

^{a}-Three measurements were conducted for each sample under 0.1 kgf load (1 kgf = 9.8 N).

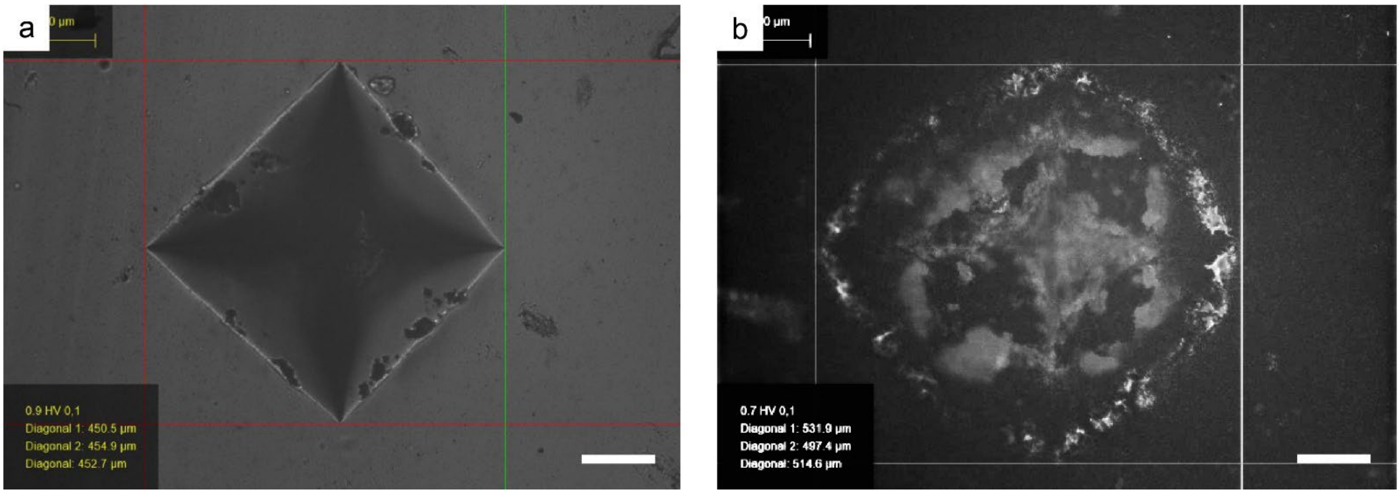

Figure S32. Micrographs of indents made on the surface of samples printed from (a) M332-40 and (b) P332-40 under 0.1 kgf load (1 kgf = 9.8 N) by Vickers hardness tester. Scale bars are 90 µm.

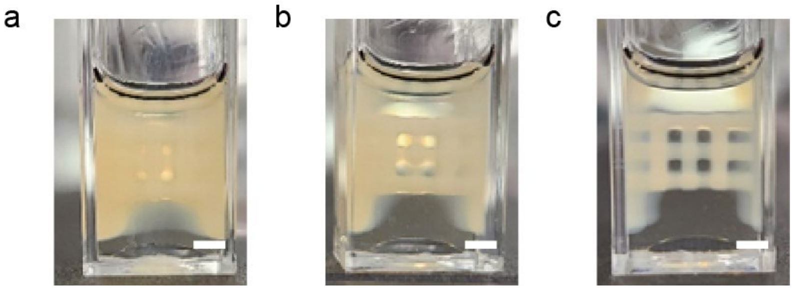

Figure S33. Optimization of printing conditions for PIPS materials prepared with P332-40 by decreasing light dose using the multi-segment model design (illustrated in the following **Notes**) in one fabrication step. (a) 2 mm min^{-1} and 11 mW mm^{-2}, 2 mm min^{-1} and 8 mW mm^{-2}, 2 mm min^{-1} and 11 mW mm^{-2}; (b) 2 mm min^{-1} and 9 mW mm^{-2}, 2 mm min^{-1} and 6 mW mm^{-2}, 2 mm min^{-1} and 8 mW mm^{-2}; (c) 2 mm min^{-1} and 8 mW mm^{-2}, 2 mm min^{-1} and 4 mW mm^{-2}, 2 mm min^{-1} and 8 mW mm^{-2}. Scale bars: 2.5 mm.

Notes: The ball-in-cage model is designed with three segments printed at various conditions in one-step fabrication.

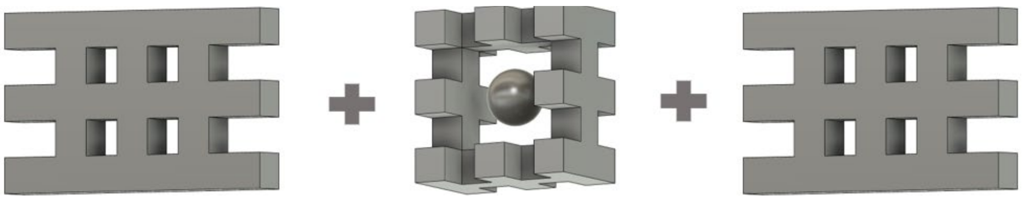

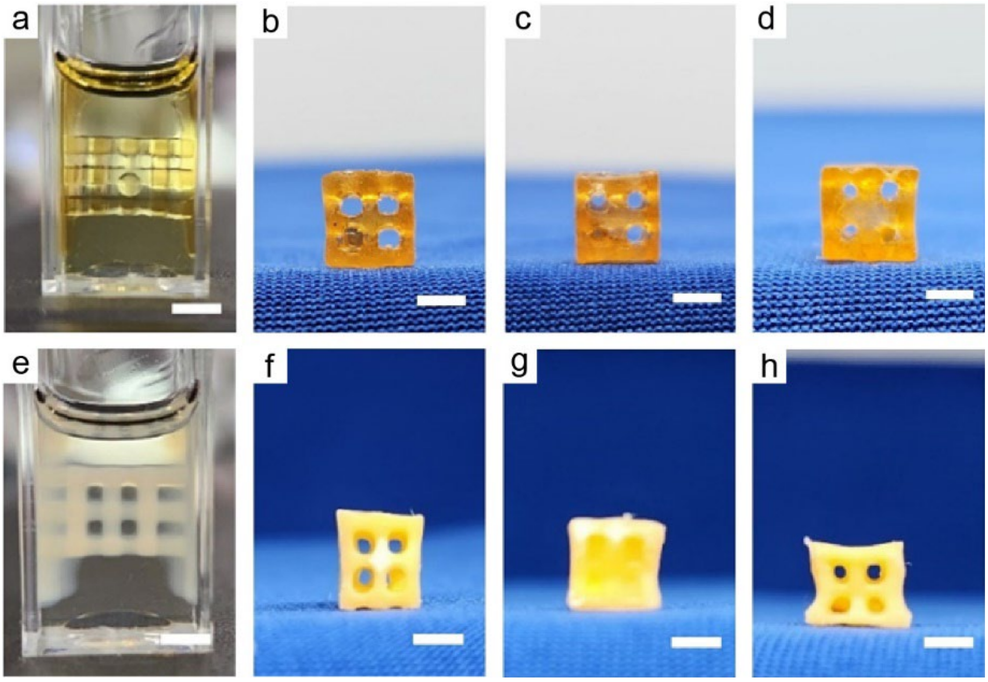

Figure S34. Photographs of fabricated objects via microphase separation and macrophase separation processes. Photos of materials printed from (a-d) M332-40 and (e-h) P332-40 formulations (a, e) before and (b-d, f-h) after post-curing, showing (b, f) the front side, (c, g) the left side and (d, h) the bottom side fabricated with optimized parameters. The PIMS object was printed under 2 mm min^{-1} and 11 mW mm^{-2}, 2 mm min^{-1} and 8 mW mm^{-2}, 2 mm min^{-1} and 11 mW mm^{-2}; The PIPS sample was printed under 2 mm min^{-1} and 8 mW mm^{-2}, 2 mm min^{-1} and 4 mW mm^{-2}, 2 mm min^{-1} and 8 mW mm^{-2}.

Supplementary References

[1] a)N. Corrigan, J. Xu, C. Boyer, X. Allonas, *ChemPhotoChem* **2019**, 3, 1193; b)T. G. McKenzie, Q. Fu, E. H. H. Wong, D. E. Dunstan, G. G. Qiao, *Macromolecules* **2015**, 48, 3864.
[2] M. Teubner, R. Strey, *J. Chem. Phys.* **1987**, 87, 3195.
[3] N. Hampu, M. A. Hillmyer, *ACS Macro Lett.* **2020**, 9, 382.
[4] P. A. Small, *J. Appl. Chem.* **1953**, 3, 71.
